# A Trypsin‐Like Serine Protease ZmNAL1a Fine‐Tunes Maize Floral Transition and Flowering Time

**DOI:** 10.1002/advs.202514635

**Published:** 2026-01-04

**Authors:** Nan Li, Qiang Ning, Zhen Li, Qian Zhang, Peilu Zhu, Jimin Zhan, Yunfu Li, Zhen Li, Liang Dong, Qing Xiong, Jiahao Liao, Jie Liu, David Jackson, Munenori Kitagawa, Zuxin Zhang, Lei Liu

**Affiliations:** ^1^ National Key Laboratory of Crop Genetic Improvement Hubei Hongshan Laboratory Huazhong Agricultural University Wuhan China; ^2^ Food Crops Institute Hubei Academy of Agricultural Sciences Wuhan China; ^3^ Yazhouwan National Laboratory Sanya China; ^4^ National Key Laboratory for Germplasm Innovation & Utilization of Horticultural Crops Huazhong Agricultural University Wuhan China; ^5^ Cold Spring Harbor Laboratory Cold Spring Harbor NY USA

**Keywords:** diffusible signal, floral transition, flowering time, shoot apical meristem, ZmNAL1a

## Abstract

The floral transition is a crucial phase in flowering plants that initiates reproductive development. Florigen, a key regulator of this transition, is expressed in the leaves and transmits environmental signals by trafficking to the vegetative shoot apical meristem, thereby promoting the floral transition. However, whether additional signals, expressed outside the meristem, control the flowering transition remains to be explored. This study identified another floral transition signal, ZmNAL1a, which encodes a trypsin‐like serine protease and can move from the leaf to the shoot apical meristem via plasmodesmata to regulate floral transition in maize. Mutation of *ZmNAL1a* suppresses the expression of key flowering genes in the shoot apical meristem, resulting in a delayed floral transition and flowering. ZmNAL1a interacts with and degrades RAMOSA1 ENHANCER LOCUS2 (REL2), a TOPLESS‐like corepressor, which can regulate the expression of flowering genes by affecting histone acetylation and transcriptional regulation alongside ZmEREBP147, an AP2/EREBP transcription factor. These findings suggest that ZmNAL1a is a diffusible signal that regulates the floral transition and flowering via a conserved NAL1‐TOPLESS epigenetic regulation module and through transcriptional regulation. This discovery broadens the understanding of flowering control, offering potential targets for improving adaptation and crop yield through precise manipulation of flowering time.

## Graphical abstract

This study identifies a trypsin‐like serine protease, ZmNAL1a, that moves from leaves to the shoot apical meristem. ZmNAL1a promotes the floral transition by degrading TOPLESS‐like corepressor REL2, which thereby enhances the expression of key flowering genes through elevating histone acetylation and relieving REL2–ZmEREBP147‐mediated transcriptional repression. Our findings reveal a novel diffusible signal that regulates flowering time.

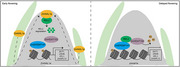

## 1 Introduction

Various intrinsic and external cues orchestrate plant growth and development, some of which are mediated by mobile signaling molecules, such as mRNAs, proteins, and small RNAs. Generally, these mobile signals are selectively transported to target cells from their synthesis domains [1]. This movement can be over a long‐distance, such as FLOWERING LOCUS T (FT) transport through the phloem from the leaf to the shoot apical meristem [2]. Some other proteins move over short distances to communicate between neighboring cells through plasmodesmata, such as the homeodomain transcriptional factor (TF) KNOTTED1 (KN1) in maize [3, 4, 5], SHOOT MERISTEMLESS (STM), BREVIPEDICELLUS (BP), TERMINAL FLOWER1 (TFL1), and WUSCHEL (WUS) in *Arabidopsis* [6, 7, 8, 9]. These cell‐to‐cell mobile signaling molecules guide plant development and response to environmental cues [1, 10, 11, 12]. For example, the proliferation of plant stem cells can be controlled by signals from neighboring cells and determinate organ primordia [10, 11, 12]. However, until now, only a limited number of mobile or diffusible factors have been characterized and functionally analyzed in plants.

In flowering plants, the floral transition marks the onset of reproductive organ development and determines the timing of flowering, beginning with the transition of the vegetative shoot apical meristem (SAM) to the inflorescence meristem (IM). SAM enlargement is regarded as a hallmark of this transition [13, 14]. During this process, signals from distant leaves trigger identity changes in meristem cells at the shoot apex [10, 15]. Florigen was the first verified long‐distance mobile signal that moves from the leaves to the shoot apex through the phloem to control the floral transition, including *Arabidopsis* FT and its homolog Hd3a in rice [2, 16, 17, 18, 19]. In *Arabidopsis*, *FT* is activated by CONSTANS (CO) in response to long‐day photoperiods, and prolonged cold exposure can alleviate the repression of *FT* expression by FLOWERING LOCUS C (FLC). FT interacts with the bZIP transcription factor FD to regulate another floral activator, *SUPPRESSOR OF OVEREXPRESSION OF CONSTANS 1* (*SOC1*) [16]. In turn, these factors trigger the expression of floral‐meristem‐identity genes, including *LEAFY* (*LFY*) and *APETALA 1* (*AP1*), leading to the floral transition [20]. Another mobile flowering signal, FLOWERING PROMOTING FACTOR 1 (FPF1)‐LIKE PROTEIN 1 (FLP1), regulates flowering time in parallel with FT, as well as affecting stem elongation during the reproductive transition [21].

However, the evidence regarding mobile or diffusible factors that control the floral transition in maize is not as extensive. The only indication comes from the maize FT homolog *Zea mays* CENTRORADIALIS8 (ZCN8), which is also suggested as a mobile flowering time signal, likely through a similar leaf‐to‐shoot apex trafficking model [22]. Many genes underlie flowering time regulation in maize, such as *Vgt1* (*Vegetative to generative transition 1*) [23], *ZmCCT9* (*CONSTANS*, *CONSTANS‐LIKE*, *TOC1*) [24], *ZmCCT10* [25], *ZmMADS4/67/69* [26, 27], *ZmSPL13/29* (*SQUAMOSA PROMOTER BINDING PROTEIN‐LIKE*) [28], as well as *DLF1* (*Delayed flowering1*, *FD* homolog) [29], *ZAG6* (*Zea mays AGAMOUS‐LIKE6*, *SOC1* homolog) [30], and *ZFL1* (*Zea mays FLORICAULA/LEAFY 1*, *LEAFY* homolog) [31]. Although these findings provide valuable insights, our understanding of other mobile or diffusible signals that might contribute to the floral transition in maize remains limited, and further studies are needed to identify additional factors and elucidate their mechanisms.

Optimized flowering time is also crucial for crops to adapt to their local environment and achieve high yields [32]. Therefore, searching for novel flowering signal molecules and revealing their diverse functions is vital to extending our understanding of plant growth and development and improving crop productivity. This study dissected flowering time regulation gene networks and identified additional signal molecules that fine‐tune the floral transition in maize, a major crop. We found a novel flowering signal molecule encoded by a trypsin‐like serine protease, ZmNAL1a, which appears to move from leaves to the shoot apex and influence the floral transition at the shoot apical meristem. ZmNAL1a has trypsin‐like serine protease activity and could degrade RAMOSA1 ENHANCER LOCUS2 (REL2), a TOPLESS‐like corepressor. ZmNAL1a could also activate key floral activators and floral meristem identity genes indirectly, by restricting REL2‐mediated histone deacetylation and transcriptional repression accompanied by ZmEREBP147.

## 2 Results

### 2.1 *ZmNAL1a* Positively Regulates the Flowering Time by Influencing the Floral Transition in Maize

The previous study constructed a multi‐omics integrative network map for maize flowering time and validated seven genes that lack evidence of a function in flowering time across plant species, including a trypsin family protein‐encoding gene, *Zm00001d026585* [33]. In addition, all four maize trypsin family protein‐encoding genes were found to participate in the flowering time regulation network [33]. We next constructed a flowering time sub‐network using all four trypsin family protein‐encoding genes and nine known flowering‐related genes, such as MADS‐box protein 1 (ZmMADS1) [34], MADS‐box protein 15 (ZMM15) [35], GIGANTEA1 (ZmGI1) [36], and DLF1 [29], with a minimal connection weight above 0.3. We found that all four trypsin family protein‐encoding genes could connect to this sub‐network, mostly through indirect interactions with known flowering time genes (Figure S1a and Table S1). However, in an association analysis of natural variants with flowering time in 540 diverse inbred lines, we found significant association signals for only one of the four trypsin family genes, *Zm00001d002323* (Figure S1b). *Zm00001d002323* is an ortholog of rice *NARROW LEAF1* (*NAL1*), which regulates rice leaf size through degradation of the TOPLESS‐related corepressor (Figures S2 and S3) [37, 38]. Thus, we referred to *Zm00001d002323* as *ZmNAL1a*, and its closest paralog (*Zm00001d026296*) as *ZmNAL1b*. The previously identified flowering time‐related trypsin family protein‐encoding gene, *Zm00001d026585* [33], was not detected in association with natural variation of flowering time, and it belongs to another subbranch on the phylogenetic tree with ZmNAL1a. Candidate gene association analysis revealed that a single‐nucleotide polymorphism (SNP) in the promoter region of *ZmNAL1a* was significantly associated with the days‐to‐anthesis (DTA) flowering time trait in 540 diverse maize inbred lines. This A/G variant, SNP‐1468, 1,468 bp upstream of the *ZmNAL1a* transcription start site, was significantly associated with DTA (*P* = 8.3 × 10^−4^) (Figure S4a), and the “A” allele significantly delayed flowering compared to the “G” allele (Figure S4b–e). These results suggest that *ZmNAL1a* may be a candidate in the maize flowering time regulation network, and *ZmNAL1a* and *ZmNAL1b* were chosen for further functional investigation.

**FIGURE 1:**
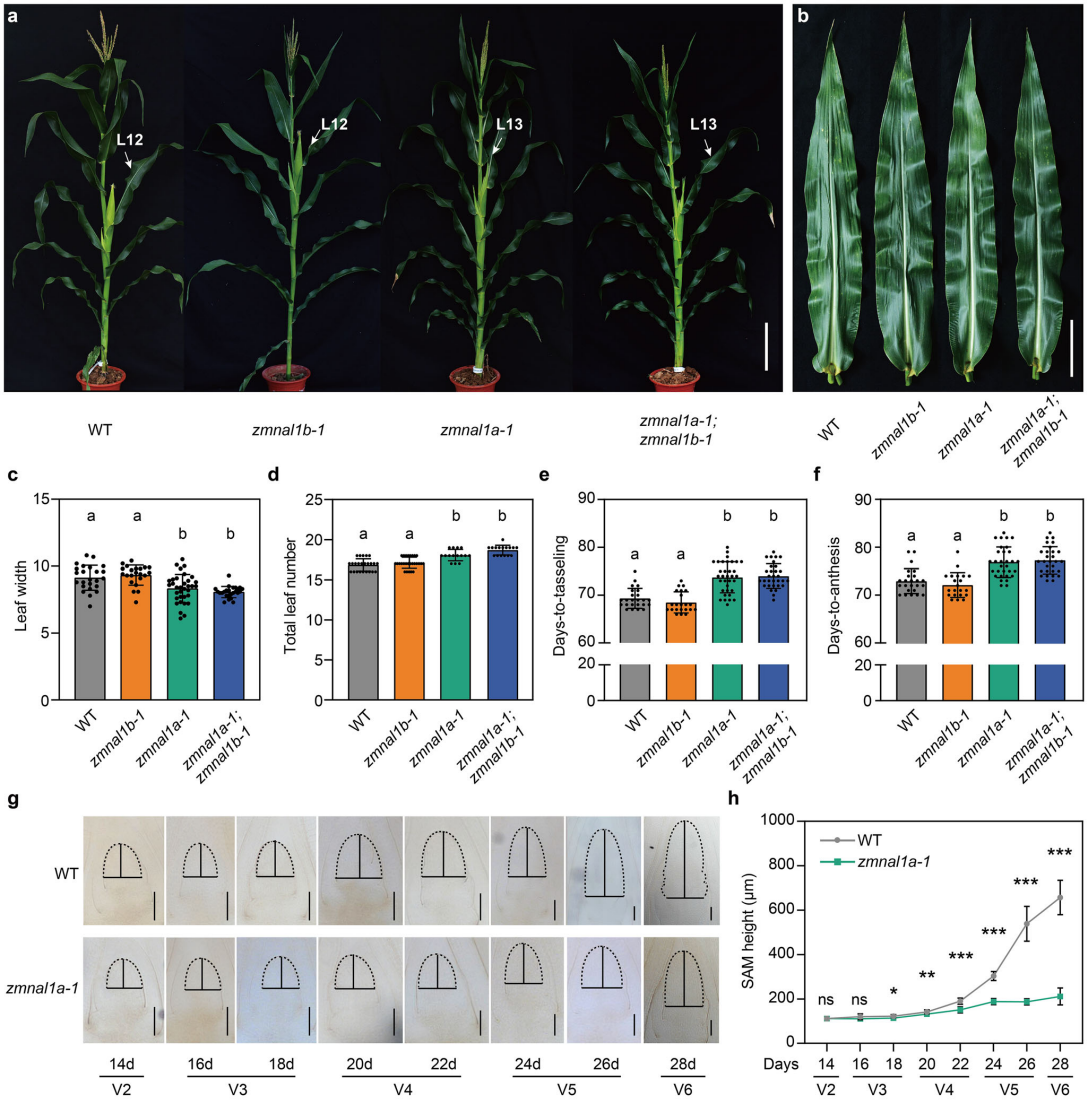
*ZmNAL1a* positively regulates flowering time in maize. (a) Morphologies of wild‐type (WT), *zmnal1b‐1, zmnal1a‐1*, and *zmnal1a‐1*; *zmnal1b‐1* mutants. White arrows indicate the maize ear leaf. L marks the leaf. Scale bars, 20 cm. (b) Comparison of leaf morphology among WT and mutant lines. Scale bars, 10 cm. (c) Phenotypic comparison of leaf width in WT (*n* = 24), *zmnal1b‐1* (*n* = 21), *zmnal1a‐1* (*n* = 34), and *zmnal1a‐1*; *zmnal1b‐1* (*n* = 25). (d) Total leaf number in WT (*n* = 24), *zmnal1b‐1* (*n* = 27), *zmnal1a‐1* (*n* = 15), and *zmnal1a‐1*; *zmnal1b‐1* (*n* = 16). (e, f) Phenotypic analysis of days‐to‐tasseling (e) and days‐to‐anthesis (f) in the WT (*n* = 24), *zmnal1b‐1* (*n* = 21), *zmnal1a‐1* (*n* = 31), and *zmnal1a‐1*; *zmnal1b‐1* mutants (*n* = 29). (g, h) *ZmNAL1a* knockout plants have smaller shoot apical meristems (SAM) compared with the corresponding WT siblings. (g) Images show cleared SAMs in the WT and *zmnal1a‐1* mutants at 14, 16, 18, 20, 22, 24, 26, and 28 days after planting, and solid lines indicate the meristem height and diameter. Scale bars, 100 µm. (h) SAM height of *zmnal1a‐1* mutants and WT (n ≥ 8). Vegetative growth stages (V stages) were defined according to the full extension of the leaf collar of the uppermost leaf. Data are presented as the mean ± s.d. In (c–f), different lowercase letters indicate significant groupings (*P <* 0.05, one‐way ANOVA, Tukey's HSD test, Table S2). In (h), significance was assessed by two‐tailed Student's *t*‐test (^*^ *P* < 0.05, ^**^ *P* < 0.01, ^***^ *P* < 0.001, Table S2). Source data are provided as a Source Data file.

To validate the function of *ZmNAL1*, we created two independent knockout mutants for *ZmNAL1a* and *ZmNAL1b* using CRISPR/Cas9 genome editing. These mutants had insertions/deletions soon after the start codon, causing frameshifts and premature stop codons, removing the serine protease domain and likely resulting in null mutants (Figure S5). Narrower leaves are one of the most striking phenotypes of rice *nal1* mutants [38]. Therefore, we measured leaf size in the *zmnal1* mutants, and observed significantly narrower leaves in *zmnal1a* single and *zmnal1a*;*zmnal1b* double mutants compared to their wild‐type (WT) sibling controls (Figure 1a–c; Figure S6a). Notably, *zmnal1a* and *zmnal1a*;*zmnal1b* mutants also had a significant increase in leaf number and internode number (Figure 1d; Figure S6b), and were delayed in days‐to‐tasseling (DTT, 4.5 days on average) and DTA (4.1 days on average) (Figure 1e,f; Figure S7). In addition, *zmnal1a*;*zmnal1b* double mutants have a slight decrease in plant and ear height (Figure 1a; Figure S6c,d), while *zmnal1b* single mutants were similar to the WT controls for these agronomic traits (Figure 1a–f; Figures S6 and S8). These phenotypes indicate that *ZmNAL1a* acts as a pleiotropic regulator of agronomic traits, especially for flowering time, similar to other delayed‐flowering mutants in altering both flowering time and leaf number [23, 26, 28].

Next, we examined the dynamic SAM‐to‐IM transition of *zmnal1a‐1* mutants and WT controls to ask if *ZmNAL1a* regulates flowering time by influencing the floral transition. The *zmnal1a‐1* mutant SAMs enlarged more slowly compared to the WT (Figure 1g,h; Figures S9 and S10). The WT seedlings had an obvious SAM‐to‐IM transition, characterized by a rapid enlargement in SAM size, at ∼ 22 days after planting (DAP) (Figure 1g,h; Figures S9 and S10). In contrast, the SAM‐to‐IM transition occurred approximately four days later, at ∼ 26 DAP in *zmnal1a‐1* seedlings (Figure 1g,h; Figures S9 and S10). This delay in timing of the floral transition matched the flowering time delay (4.1‐4.5 days for DAT and DTT, Figure 1e,f; Figure S7) in *zmnal1a‐1*, suggesting that the delayed floral transition is a significant reason for its delayed flowering. This result demonstrates that *ZmNAL1a* ensures a prompt floral transition, and its loss of function results in delayed IM initiation and flowering.

### 2.2 ZmNAL1a Acts as a Diffusible Signal That Moves from Leaf Primordia to the Shoot Apical Meristem

**FIGURE 2:**
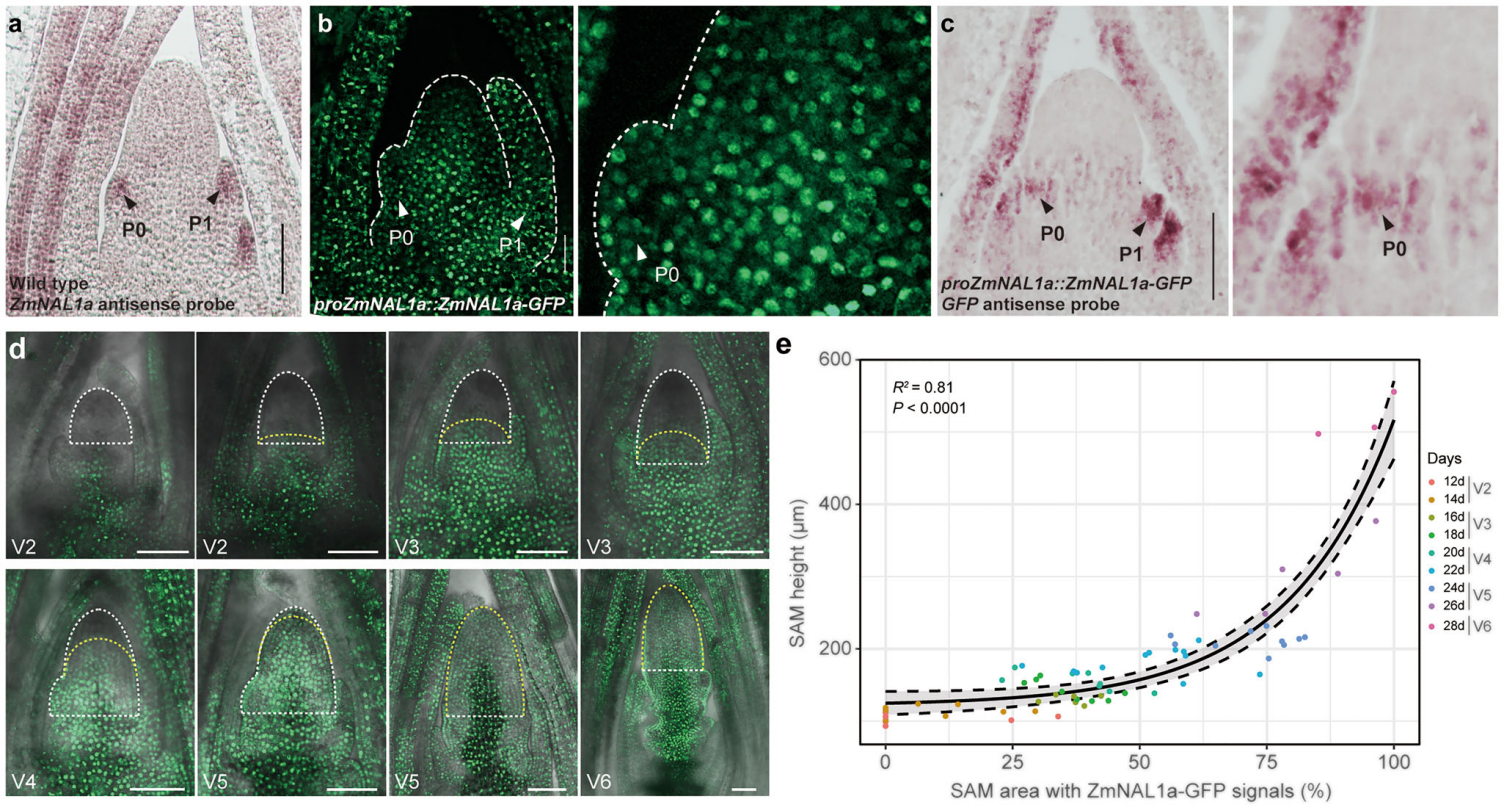
Expression of *ZmNAL1a* and dynamic distribution of ZmNAL1a‐GFP in the SAM. (a) RNA in situ hybridization indicated that *ZmNAL1a* mRNA is enriched in the leaf primordia but not in the SAM. Black arrowheads indicate P0 and P1 leaf primordia. Scale bars, 100 µm. (b) ZmNAL1a protein distribution in the shoot apex of *proZmNAL1a*::*ZmNAL1a‐GFP* plants and magnification of the P0 region. White arrowheads indicate P0 and P1. Scale bars, 50 µm. (c) RNA in situ hybridization of *ZmNAL1a*‐*GFP* using *GFP* antisense probes in the shoot apex of *proZmNAL1a*::*ZmNAL1a*‐*GFP* plants. Black arrowheads indicate P0 and P1. Scale bars, 50 µm. (d) Spatiotemporal dynamics of ZmNAL1a‐GFP distribution in the SAM. GFP fluorescence is shown in green. The white dashed lines indicate the outline of the SAM, while the yellow dashed lines outline the region with detectable ZmNAL1a‐GFP signals. Scale bars, 100 µm. (e) Correlation between the proportion of SAM area showing ZmNAL1a‐GFP signals (%) and SAM height (µm) at different developmental stages (12–28 days after planting). Each dot represents a single SAM; color indicates the corresponding time point. The black curve shows a nonlinear regression fit (*R* ^2^ = 0.81, *P* < 0.0001). Vegetative growth stages (V stages) were defined according to the full extension of the leaf collar of the uppermost leaf. Source data are provided as a Source Data file.

*ZmNAL1a* was predominantly expressed in the developing shoot apex (Figure S11a). We therefore examined its spatiotemporal expression pattern to understand its function in the floral transition. *ZmNAL1a* mRNA was enriched in maturing and incipient leaf primordia, but strikingly absent from the SAM itself (Figure 2a; Figure S12a,c,d). In contrast, ZmNAL1a protein accumulated both in the leaf primordia and in the center of the SAM, as illustrated by green fluorescent protein (GFP) signals in *proZmNAL1a::ZmNAL1a‐GFP* transgenic lines, which express ZmNAL1a‐GFP fusion proteins driven by the *ZmNAL1a* native promoter (Figure 2b). We also performed RNA in situ hybridization in *proZmNAL1a::ZmNAL1a‐GFP* lines using a GFP antisense probe, and found *ZmNAL1a‐GFP* mRNA was enriched in leaf primordia (Figure 2c; Figure S12b–d), similar to the native *ZmNAL1a* mRNA (Figure 2a). Therefore, the ZmNAL1a‐GFP signal in the center of the SAM is unlikely due to mis‐expression of the *proZmNAL1a::ZmNAL1a‐GFP* transgene, and the distribution of the ZmNAL1a‐GFP protein appeared to have extended outside its mRNA expression domain into the center of the SAM.

We further tracked the dynamic distribution of ZmNAL1a‐GFP protein in the shoot apex, and its relationship with the SAM‐to‐IM transition (Figure 2d,e). We first conducted a complementation assay to validate the biological function of ZmNAL1a‐GFP by introducing the *proZmNAL1a*::*ZmNAL1a‐GFP* transgene into the *zmnal1a‐1* knockout line (Figure S13). The *proZmNAL1a*::*ZmNAL1a‐GFP* transgene could largely complement the delayed flowering phenotype of *zmnal1a‐1* mutants (Figure S13). This result suggests that native *ZmNAL1a‐GFP* expression retains the biological function of *ZmNAL1a* in regulating flowering time. We next checked ZmNAL1a‐GFP expression in shoot apical regions, and measured SAM size at each stage in the *proZmNAL1a*::*ZmNAL1a‐GFP*;*zmnal1a‐1* line (Figure 2d,e). In 12 DAP seedlings, we observed clear ZmNAL1a‐GFP signals in the leaf and the stem tissues in the shoot apex, but they were excluded from the SAM (Figure 2d). ZmNAL1a‐GFP was expressed in a gradient, with a stronger signal at the base and a weaker signal higher up close to the SAM at 12 DAP. This gradient appeared to transition toward the upper region of the shoot apex after 14 DAP (Figure 2d), and later occupied almost the entire SAM/IM regions after 22 DAP (Figure 2d,e). Importantly, the height of the SAM was significantly and positively correlated with the percentage of the SAM area occupied by ZmNAL1a‐GFP (*R* ^2^ = 0.81, Figure 2e). It appeared that the SAM started to enlarge rapidly once the ZmNAL1a‐GFP signals extended to occupy ∼ 50% of the SAM area (Figure 2e). Meanwhile, the *ZmNAL1a‐GFP* mRNA was only found to be enriched in leaf primordia during these stages (Figure S12e–h). Therefore, ZmNAL1a protein appeared to travel into the SAM region from the maturing and younger leaf primordia, where it influences the SAM‐to‐IM transition after reaching the center of the SAM.

**FIGURE 3:**
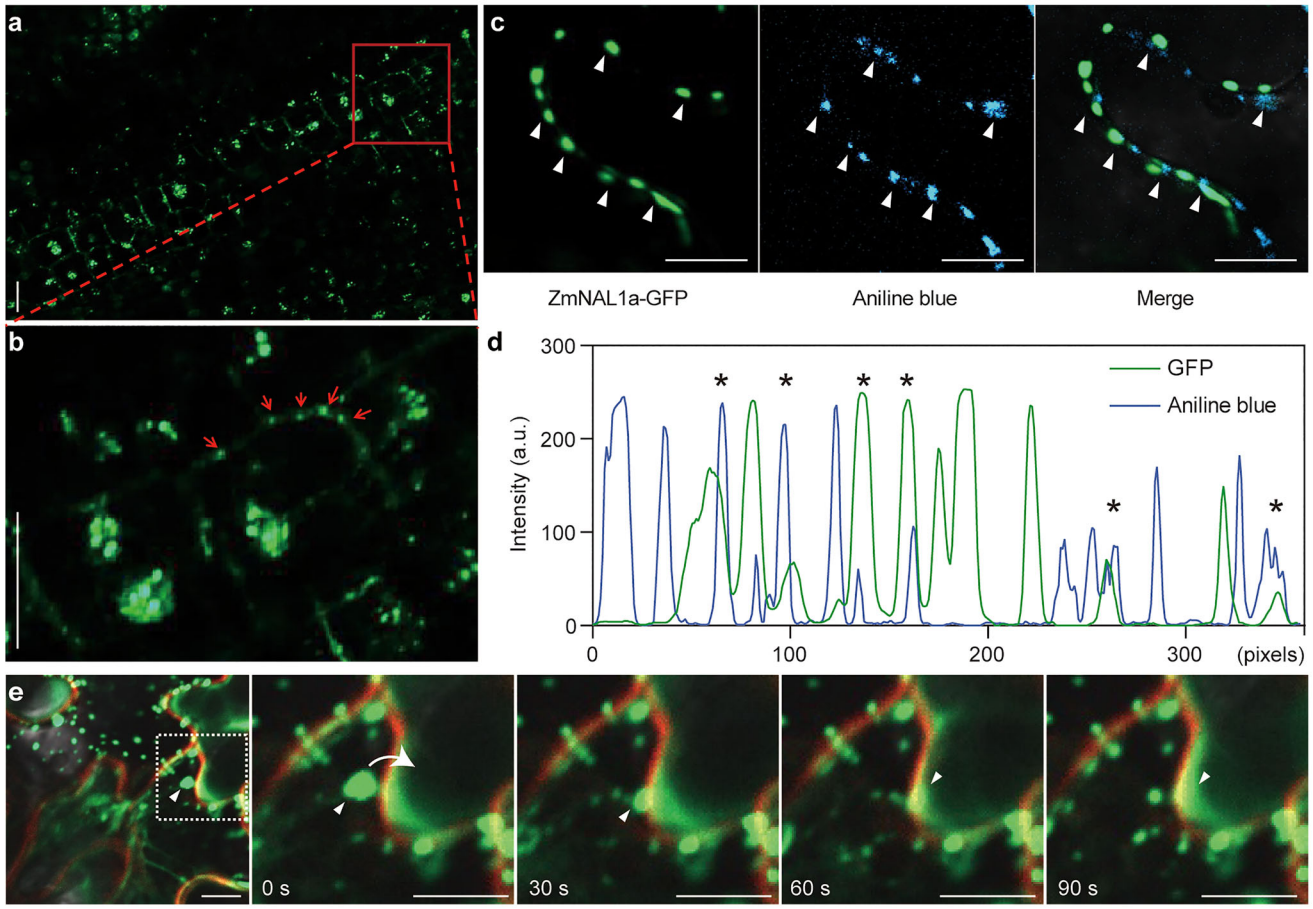
ZmNAL1a protein accumulates in puncta and moves via plasmodesmata. (a) Distribution of ZmNAL1a‐GFP fusion proteins expressed in leaves of *proZmNAL1a::ZmNAL1a‐GFP* plants. Scale bars, 20 µm. (b) An enlarged view of the boxed region in panel (a). The red arrowheads indicate punctate structures at the cell periphery. Scale bars, 20 µm. (c) ZmNAL1a‐GFP (green) accumulated in puncta at the boundary of two epidermal cells and colocalized with aniline blue staining of the plasmodesmata (arrowheads). Scale bars, 10 µm. (d) Quantitative analysis of ZmNAL1a‐GFP and aniline blue fluorescent signals. Green and blue lines show the fluorescence intensities of GFP and aniline blue along the cell‐cell boundaries, respectively, and asterisks represent overlaps. a.u., arbitrary units. (e) Representative confocal images showing the subcellular localization of ZmNAL1a‐GFP in tobacco leaf epidermal cells. Time‐lapse images of the enlarged boxed region (0, 30, 60, and 90 s). Green, GFP; Red, FM4‐64. White triangles indicate mobile puncta of the ZmNAL1a‐GFP protein. White arrows indicate the direction of movement. Scale bars, 10 µm. Source data are provided as a Source Data file.

Next, we analyzed the subcellular localization of ZmNAL1a to ask how it might move between cells. The ZmNAL1a‐GFP signal was detected in the nucleus and cytoplasm in maize leaf protoplasts (Figure S11b) and *proZmNAL1a::ZmNAL1a‐GFP* transgenic lines (Figure 3a,b). At higher magnification, ZmNAL1a‐GFP was observed in plasmodesmata‐like puncta at the periphery of leaf cells adjacent to the SAM (Figure 3a,b). To confirm this localization, we transiently expressed ZmNAL1a‐GFP in tobacco leaves and found colocalization with aniline blue‐stained plasmodesmata (Figure 3c,d). We also observed ZmNAL1a‐GFP signals near the boundaries of neighboring tobacco leaf cells, forming puncta in the cytoplasm (Figure 3e; Figures S14 and S15). Similar ZmNAL1a‐GFP puncta could also be observed in maize leaf cells (Figure S11c,d). Interestingly, some of the ZmNAL1a‐GFP puncta appeared to move to the membrane and stop (Figure 3e; Figures S14 and S15), while some of them appeared to move into neighboring cells (Figure 3e; Figures S14 and S15). These results suggest that the ZmNAL1a protein moves between cells from leaf primordia to the SAM through plasmodesmata to initiate the floral transition.

### 2.3 ZmNAL1a Targets RAMOSA1 ENHANCER LOCUS2 for Degradation

ZmNAL1a mobility may affect the floral transition by degrading downstream target proteins after moving into the SAM. Therefore, we screened for ZmNAL1a targets using immunoprecipitation followed by mass spectrometry (IP‐MS) with shoot apex tissues from *proZmNAL1a::ZmNAL1a‐GFP* transgenic plants, and identified 698 candidate interacting proteins (Table S3). Of them, 572 proteins have more than one peptide detected from IP‐MS, and were not detected in a negative control IP using non‐transgenic sibling tissue. We then selected 20 of them to validate their interactions with ZmNAL1a using BiFC and found that 65% (13/20) showed interaction signals, suggesting that most of the IP‐MS targets likely interact with ZmNAL1a (Figure S16). Furthermore, 45 of the candidate interacting proteins were present in the ZmNAL1a‐connected flowering time sub‐network (Table S3).

**FIGURE 4:**
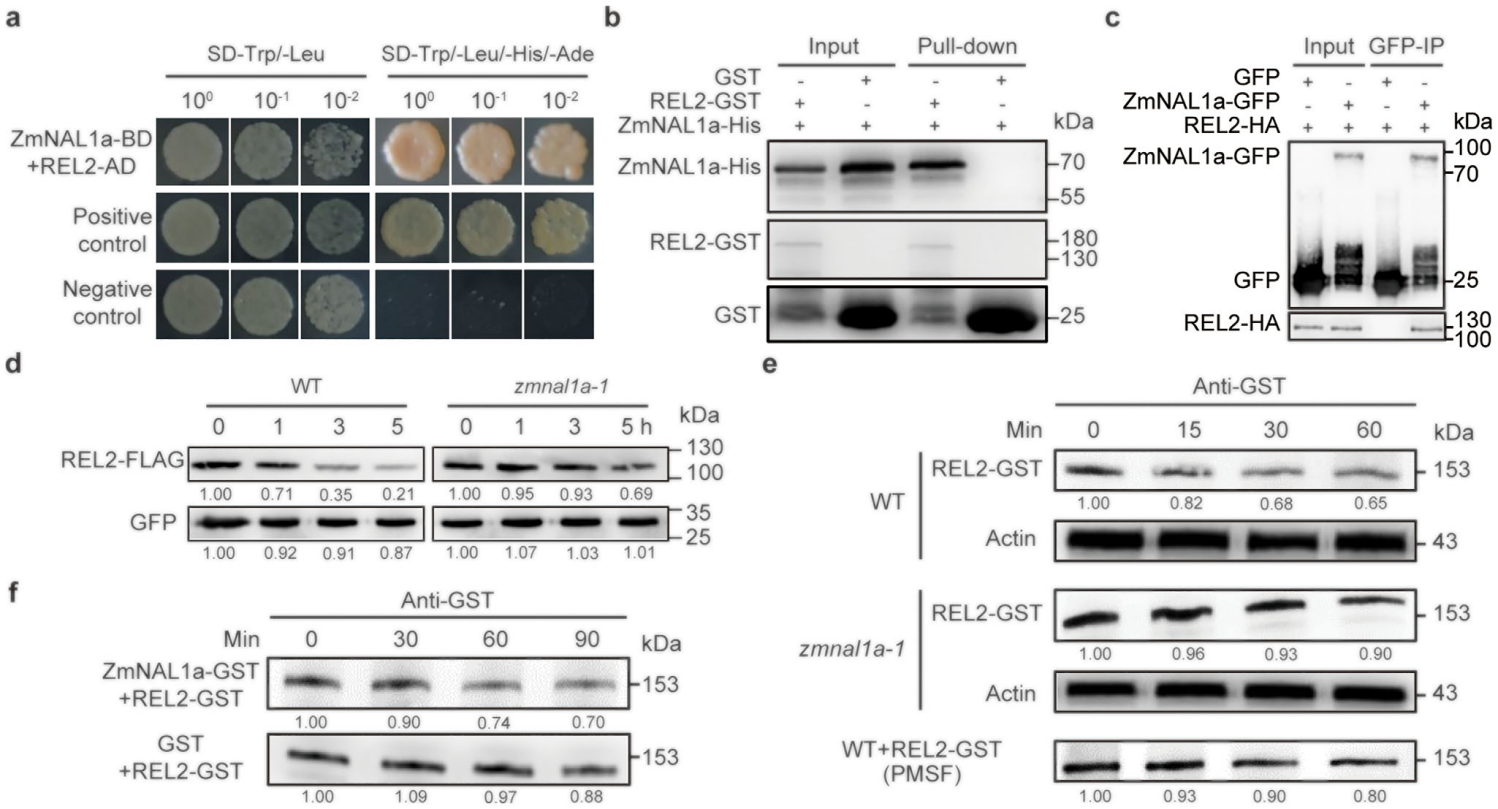
ZmNAL1a targets TOPLESS‐like corepressor REL2 for degradation. (a) Yeast two‐hybrid assay showing the interaction between ZmNAL1a and REL2. The empty vector pGADT7 was used as a negative control. (b) In vitro pull‐down assay showing the interaction between ZmNAL1a and REL2. (c) Interaction between ZmNAL1a and REL2 in tobacco leaves by co‐immunoprecipitation assay. (d, e) REL2 is more stable in *zmnal1a* than in WT, as observed from a protein expression assay in protoplasts (d) and cell‐free protein degradation assay using plant extracts (e). For e, the cell‐free protein degradation assay was conducted using WT, *zmnal1a* mutant plant extracts, and WT supplemented with 5 mm phenylmethanesulfonyl fluoride (PMSF). An equal volume of protein mixture was collected at different time points for immunoblotting detection of REL2. (f) Degradation of GST‐tagged REL2 protein after incubating with ZmNAL1a protein at different time points in vitro.

Among the candidate ZmNAL1a IP‐MS targets, TOPLESS (TPL) family proteins, such as RAMOSA1 ENHANCER LOCUS2 (REL2), were of interest because they regulate SAM development [39]. We confirmed the physical interaction between ZmNAL1a and REL2 using yeast two‐hybrid (Y2H), pull‐down, and co‐immunoprecipitation (Co‐IP) assays (Figure 4a–c). The first EAR‐like motif in the C‐terminal region of ZmNAL1a was essential for the ZmNAL1a‐REL2 interaction (Figure S17). Therefore, the NAL1‐TPL interaction was conserved between rice and maize.

Next, we performed in vivo protein degradation assays using maize protoplasts, as well as cell‐free in vitro protein degradation assays to ask if ZmNAL1a could degrade REL2. In vivo protein degradation assays found that the amount of REL2‐FLAG degradation was lower in *zmnal1a‐1* mutant protoplasts (∼31%), compared to ∼79% in WT protoplasts (Figure 4d; Figure S18a,b). Our in vitro protein degradation assays similarly found that REL2‐GST was degraded ∼3.5‐fold slower in extracts of *zmnal1a‐1* mutants compared to WT extracts. REL2‐GST degradation was also significantly inhibited by the addition of phenylmethanesulfonyl fluoride, a serine and cysteine protease inhibitor (Figure 4e; Figure S18c,d). REL2‐GST protein was also degraded by purified ZmNAL1a‐GST protein, but not with GST alone (Figure 4f; Figure S18e,f). These findings demonstrate that ZmNAL1a interacts with and degrades REL2.

### 2.4 ZmNAL1a‐REL2‐ZmHDT102 Modulates the Histone Modification State of Key Flowering Genes

**FIGURE 5:**
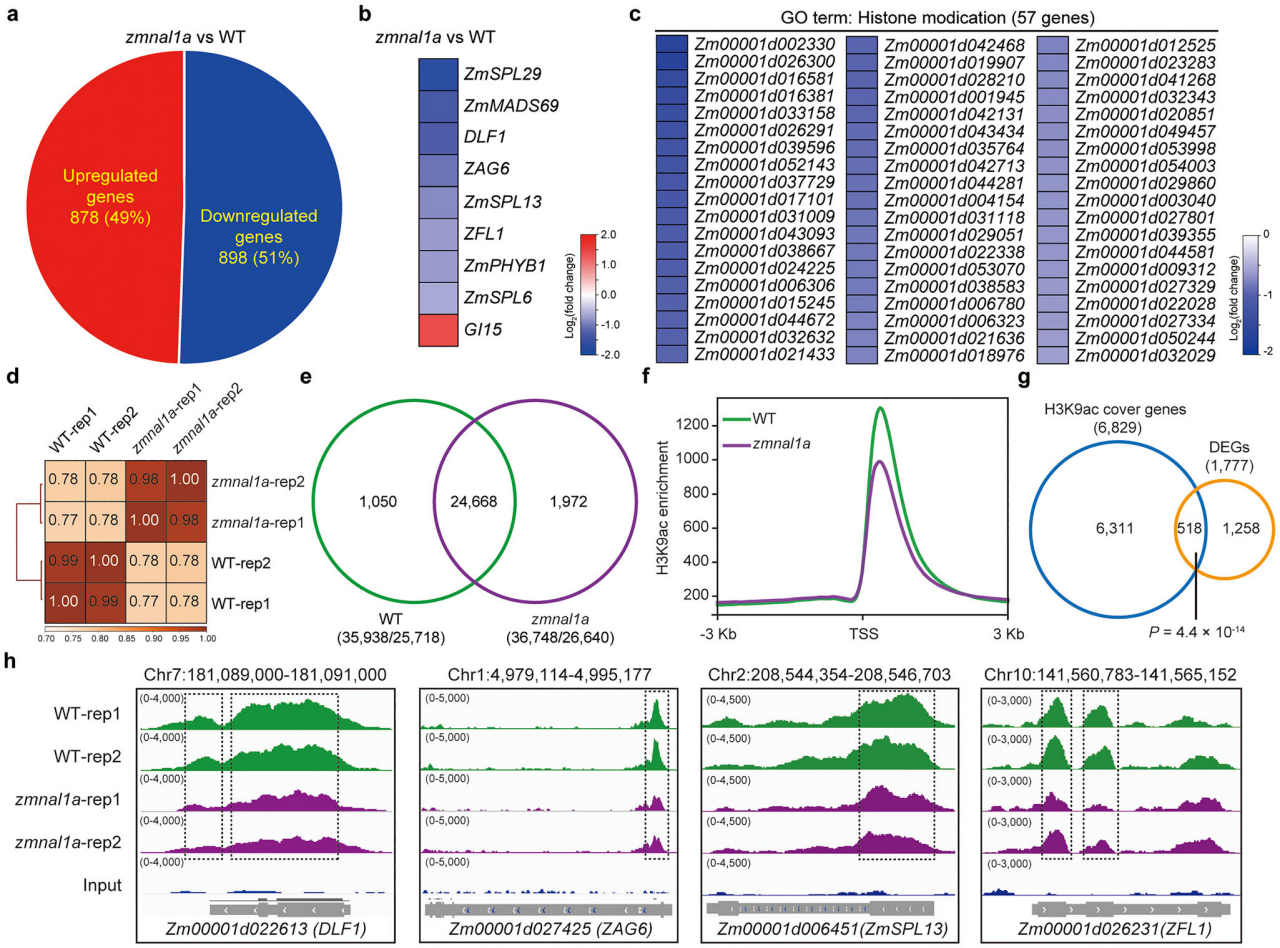
Genome‐wide analysis of transcriptome and H3K9Ac in the shoot apex of *zmnal1a* mutants and WT plants. (a) Differentially expressed genes (DEGs) detected by RNA sequencing in the shoot apex between *zmnal1a* mutants and corresponding WT siblings under fold‐change > 1.5 and FDR < 0.05. (b) Heatmap shows that eight out of nine maize flowering‐related genes were significantly downregulated in the *zmnal1a* knockout mutants. (c) Expression fold change heat maps of the GO term (GO:0016570) involved in histone modifications. (d) Pearson's correlation coefficients of H3K9Ac ChIP‐seq replicates. (e) H3K9Ac marked peak/gene numbers identified in *zmnal1a* mutants and WT plants. Two replicates of ChIP‐seq were merged in peak calling for each genotype. (f) Metaplot of the H3K9Ac level at all marked genes in *zmnal1a* mutants compared with the WT. (g) Venn diagram showing the number of overlapped genes with changed H3K9Ac and expression levels in *zmnal1a* mutants compared with WT. The *P*‐value was calculated using the hypergeometric test. (h) Integrative Genomics Viewer snapshots of H3K9Ac alterations for flowering genes: *DLF1*, *ZAG6*, *ZmSPL13* and *ZFL1*.

TOPLESS proteins are transcription corepressors that recruit histone deacetylases to mediate the epigenetic modifications of downstream genes [40, 41, 42]. We found two histone deacetylases in the ZmNAL1a IP‐MS target list (Table S3). Of them, Histone deacetylase102 (ZmHDT102) interacted weakly with ZmNAL1a; however, ZmNAL1a might not degrade ZmHDT102 (Figure S19). ZmHDT102 also interacted physically with REL2 (Figure S20). REL2 may therefore mediate a ZmNAL1a‐REL2‐ZmHDT102 complex, which could regulate genome‐wide histone acetylation levels. Consequently, we used a multi‐omic strategy to explore the molecular mechanism of ZmNAL1a in regulating the floral transition by integrating transcriptomic and histone acetylation chromatin immunoprecipitation sequencing (ChIP‐seq) analyses using developing shoot apex tissues of *zmnal1a‐1* mutants and WT plants. We identified 1,776 differentially expressed genes (DEGs), including 878 upregulated (49%) and 898 downregulated genes (51%), between *zmnal1a* mutants and WT from the transcriptomic analysis (Figure 5a; Table S4). The up‐regulated genes were primarily in basal metabolic and response to environmental cues related GO categories, such as small‐molecule and organic acid biosynthesis, response to light stimulus, and to heat, suggesting these basal metabolic‐related DEGs may contribute to ZmNAL1a function in regulating flowering time and other traits (Figure S21a). In contrast, the down‐regulated genes were significantly enriched in biological processes related to meristem development, such as reproductive and shoot growth, cell division, chromatin assembly, and histone modification (Figure S21b). Notably, 57 histone modification‐related genes (GO:0016570), including histone deacetylase and methylation‐related genes, were down‐regulated in *zmnal1a* mutants (Table S5). Eight of the nine flowering‐time‐related DEGs were also down‐regulated in *zmnal1a* mutants (Figure 5b,c). These results imply a genome‐wide alteration of histone modifications in *zmnal1a* relative to WT, which might modulate the expression of flowering‐time‐related genes.

We next performed ChIP‐seq analysis of H3K9Ac histone modification, and identified 35,938 and 36,748 H3K9Ac‐marked peaks in WT and *zmnal1a‐1* mutants, respectively (Figure 5d,e). Overall, the genic H3K9Ac levels were decreased in the *zmnal1a* mutants (Figure 5f), and 98% of the differentially acetylated genes (DAGs, *n* = 6829) were down‐regulated (*n* = 6708) (Table S6). Furthermore, ∼29% (518 out of 1,778) of the DEGs had differential acetylation in their genic region, indicating that DAGs were significantly enriched in these DEGs (*P* = 4.4 × 10^−14^, Figure 5g). Importantly, the H3K9Ac levels and expression of several essential flowering genes involved in SAM development and floral transition, including *DLF1*, *ZAG6*, *ZmSPL13*, and *ZFL1*, were significantly decreased in *zmnal1a* mutants compared to WT (Figure 5b,h). These results suggest that the ZmNAL1a‐REL2‐ZmHDT102 complex plays a role in regulating the histone modification state of key flowering genes and fine‐tunes the expression of these genes to influence the floral transition. Our results explain the delay in floral transition and late flowering in *zmnal1a* mutants.

### 2.5 ZmEREBP147 Represses Flowering Gene Expression by Interacting with REL2

**FIGURE 6:**
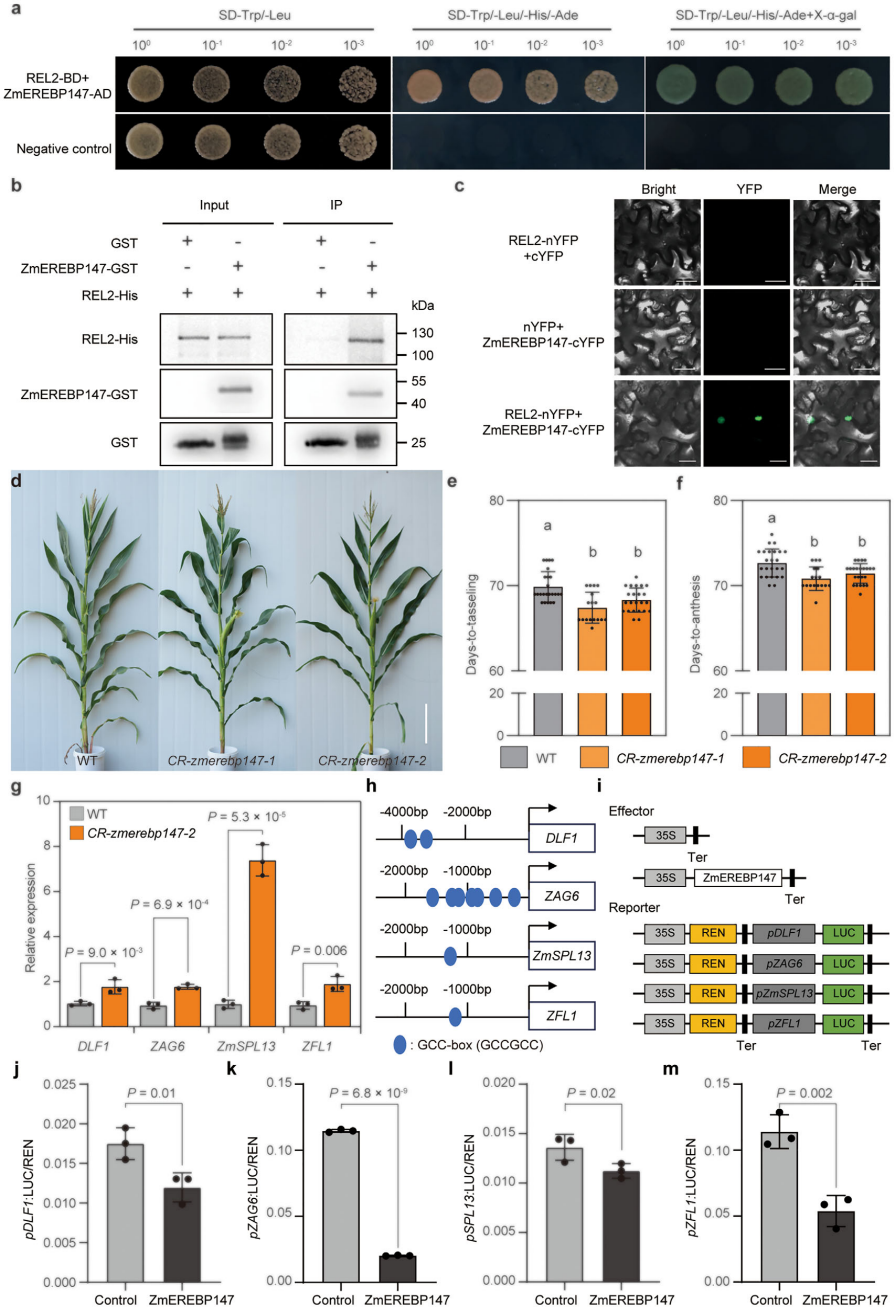
ZmEREBP147 interacts with REL2 and represses the expression of flowering genes. (a) Y2H assay of ZmEREBP147 showing interaction with REL2. The empty vector pGADT7 was used as a negative control. (b) In vitro pull‐down assays showing the interaction between ZmEREBP147 and REL2. ZmEREBP147‐GST or GST alone was incubated with REL2‐His and purified using glutathione‐agarose beads, followed by immunoblotting with anti‐GST and anti‐His antibodies. (c) Bimolecular fluorescence complementation analysis of the interaction between ZmEREBP147 and REL2 in tobacco leaves. Scale bars, 20 µm. (d) Morphology of WT and *zmerebp147* mutant plants. Scale bars, 10 cm. (e, f) Phenotypic analysis of DTT (e) and DTA (f) in the WT (*n* = 24), *CR‐zmerebp147‐1* (*n* = 17), and *CR‐zmerebp147‐2* lines (*n* = 23). (g) Relative expression levels of flowering time genes in WT and *zmerebp147* (*n* = 3). h‐m) ZmEREBP147 could bind to the promoters of key flowering time genes and repress their expression. (h) Schematic diagram of the *DLF1*, *ZAG6*, *ZmSPL13* and *ZFL1* promoters. Blue dots indicate the putative GCC‐box motif. (i) The schematic diagram shows the effectors and reporter used in the transient transcriptional activity assays. j‐m) ZmEREBP147 represses the expression of *DLF1*, *ZAG6*, *ZmSPL13* and *ZFL1*. Data are presented as means ± s.d., from three biological replicates. In (e‐f), different lowercase letters indicate significant groupings (*P* < 0.05, one‐way ANOVA, Tukey's HSD test, Table S2). In (g, j–m), significance was assessed by a two‐tailed Student's *t*‐test. Exact *P* values are shown. Source data are provided as a Source Data file.

REL2 physically interacts with the transcription factor ZmEREBP147 [39]. *REL2* and *ZmEREBP147* have similar expression patterns in the SAM (Figure S22), and REL2‐ZmEREBP147 interaction was verified using Y2H, pull‐down, and bimolecular fluorescence complementation (BiFC) assays (Figure 6a–c). Thus, we asked if ZmEREBP147 regulates the expression of flowering genes. *ZmEREBP147* knockout mutants were created using CRISPR/Cas9 genome editing (Figure S23). *zmerebp147* mutants had an earlier SAM‐to‐IM transition and flowering than their WT siblings (Figure 6d–f; Figure S24). The expression levels of some known flowering genes, including *DLF1*, *ZAG6*, *ZmSPL13*, and *ZFL1*, were also significantly increased in *zmerebp147* mutants (Figure 6g). In addition, ZmEREBP147 could bind its promoter regions to repress their transcription (Figure 6h–m), supporting the hypothesis that ZmEREBP147 functions as a transcriptional repressor of flowering genes. Together, our results suggest that ZmNAL1a tightly regulates the floral transition through a REL2‐ZmEREBP147 module that mediates transcriptional regulation of essential flowering genes.

## 3 Discussion

**FIGURE 7:**
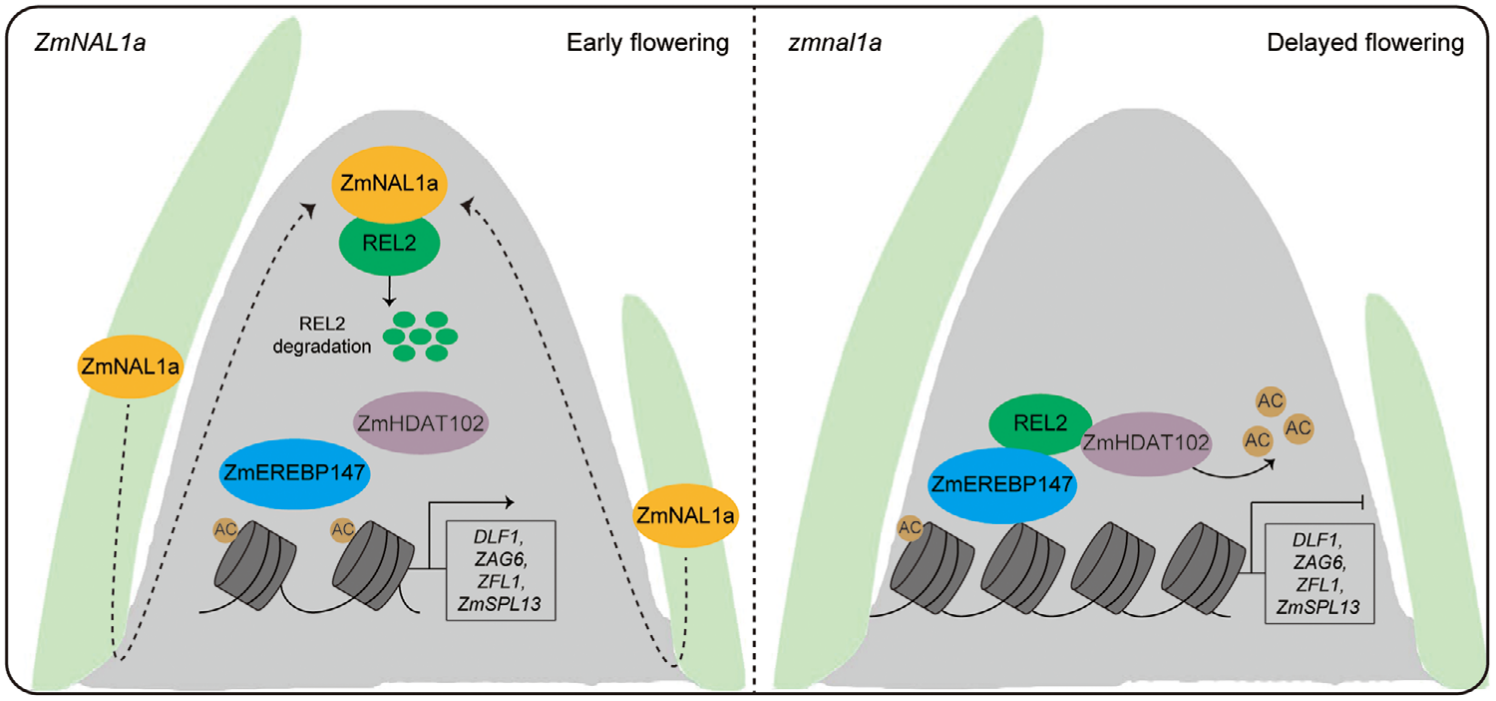
Proposed model for the function of *ZmNAL1a* in promoting maize floral transition. ZmNAL1a moves from leaf primordia to the SAM through plasmodesmata, interacting with and degrading the corepressor REL2. This prevents H3K9 deacetylation in essential flowering genes and disrupts the interaction between REL2 and ZmEREBP147, a flowering gene repressor. Consequently, this mechanism regulates floral transition and flowering time in maize. The dotted arrows indicate the cell‐to‐cell transmission of ZmNAL1a protein. AC, acetylation.

Numerous studies show that mobile signals, particularly florigen, control flowering in model plants such as *Arabidopsis* and rice. A florigen homolog, ZCN8, and many other flowering genes have been identified in maize, and flowering molecular pathways have been systematically uncovered [22, 33]. However, our understanding of the detailed molecular mechanisms for the signaling molecules, especially mobile or diffusible factors, in controlling flowering is poor [1, 43]. Our study suggests that ZmNAL1a is a new diffusible factor that traffics from mature and immature leaves to the SAM to promote the floral transition in maize. ZmNAL1a interacts with and degrades its corepressor REL2 in the SAM to prevent H3K9 deacetylation in key flowering genes, allowing them to be expressed and to promote the floral transition. Additionally, REL2 degradation by ZmNAL1a can affect the interaction between REL2 and ZmEREBP147, which represses the expression of flowering genes, thereby regulating floral transition and flowering time (Figure 7). Our study suggests that ZmNAL1a, a novel candidate non‐cell autonomous factor in maize, regulates the SAM‐to‐IM transition and flowering time, and provides a novel mechanism for controlling the identity of meristem cells by moderating histone modification and expression of flowering genes (Figure 7).

Rice *NAL1* has pleiotropic biological functions, including regulating plant height, leaf morphology, root architecture, photosynthesis rate, chlorophyll content, and grain yield [44, 45, 46]. The rice *nal1* mutants have obvious defects in leaf width and plant height. These functions depend on an NAL1–OsTPR2 module that moderates histone acetylation and expression levels of hormone signaling genes, such as auxin and strigolactone [38]. Our study similarly observed narrow leaf and short stature phenotypes in *zmnal1a* mutants, and *ZmNAL1a* could partially complement rice *nal1* mutants [38]. Therefore, *ZmNAL1a* is likely active in maize seedling tissues, such as the leaf, stem, and SAM, and is involved in similar hormone signaling pathways to regulate leaf size and plant height, suggesting a conserved function of this plant‐specific serine protease. Furthermore, our results reveal a novel function of *NAL1* in fine‐tuning the floral transition and flowering time by indirectly regulating the expression of floral transition‐related genes in maize. The conserved NAL1‐TPL‐HDAC epigenetic regulation module and the newly validated NAL1‐TPL‐ZmEREBP147 transcriptional regulation module together may underlie *ZmNAL1a* function in control of maize flowering (Figure 7). However, other pathways, such as basal metabolism and hormone signaling [38, 44, 45, 46], could also contribute to flowering time regulation by ZmNAL1a. Further analysis is needed to ask whether the flowering regulation function of *NAL1* is conserved in other crop species.

A series of flowering genes genetically controls the timing of the floral transition in angiosperms. In *Arabidopsis*, the expression of two central flowering time regulators, FLC and FT, was controlled by various chromatin modifications [47]. For example, activation of the flowering repressor FLC is associated with multiple histone modifications, such as acetylation, methylation, monoubiquitination, etc., whereas various repressive histone modifications silence FLC [43, 47]. In *Arabidopsis* and some cereals, which need vernalization to trigger flowering, low temperature can induce epigenetic silencing of FLC through repressive histone modifications [43, 47]. The loss of FLC will release its repression of *FD*, *FT*, and *SOC1*, thereby activating floral meristem identity genes, such as *LEAFY*, to promote floral transition and flowering [43, 47]. In maize, a non‐vernalizing plant, our study found that the expression of key flowering genes, such as *DLF1*, *ZAG6*, and *ZFL1*, homolog genes of *FD*, *SOC1*, and *LEAFY* in *Arabidopsis*, respectively, is also regulated by histone modifications. Our findings show that the default histone state in maize for these key flowering genes during the floral transition is an active form, with acetylation modifications. The flowering signal identified here, ZmNAL1a, acts as a brake signal for histone deacetylation in these key flowering genes, mediated by REL2‐ZmHDT102. Our results show that epigenetic regulation of flowering genes is a common molecular mechanism underlying the floral transition and flowering time regulation. Plant species maintain diverse chromatin‐modifying targets during evolution. Therefore, fine‐tuning flowering time‐specific chromatin‐modifying components may improve crop flowering traits by reshaping the epigenetic status of target loci.

The shoot apical meristem is usually hidden in several layers of leaf primordia. However, it can connect with the environment through the vascular system and intercellular transport. Thus, the shoot apical meristem can respond to environmental cues [20, 22]. Signals such as FT and Hd3a protein translocate from the leaf to the shoot apical meristem through these connection systems, promoting the SAM to floral meristem transition [18]. Our study observed a dramatically uneven distribution of ZmNAL1a mRNA and protein in leaf primordia and the SAM, with almost no mRNA but considerable protein accumulation in the SAM. Moreover, ZmNAL1a localizes at plasmodesmata and moves between cells through these channels, and ZmNAL1a appeared to move into the SAM from leaves. Therefore, it is likely that the ZmNAL1a protein moves from the leaf to the center of the SAM to maintain the expression of genes that affect floral transition.

However, whether other molecules assist the ZmNAL1a protein in its cell‐to‐cell trafficking is unknown. Further studies are needed to confirm ZmNAL1a movement and understand the mechanism of its intercellular transport. It is also unclear what kinds of signals, such as environmental cues or internal developmental signals, ZmNAL1a could transmit. Moreover, genetic evidence is needed to confirm the molecular mechanism of ZmNAL1a in regulating flowering time through REL2 and ZmEREBP147. In summary, this study characterizes a novel molecular signal in crop flowering, broadens our understanding of flowering regulation, and offers potential targets for improving adaptation and crop yield through precise manipulation of flowering time.

## 4 Experimental Section

### 4.1 Phenotypic Analysis and Plant Materials

CRISPR‐Cas9 was used to generate null alleles of *ZmNAL1a*, *ZmNAL1b*, and *ZmEREBP147*. Single‐guide RNAs (sgRNAs) were designed for these genes using the CRISPR‐P 2.0 website (http://cbi.hzau.edu.cn/crispr/) [48, 49]. A CPB binary vector was used to perform these sgRNA arrays as CRISPR genome‐editing constructs. The 2‐kb *ZmNAL1a* native promoter was cloned, linked to *ZmNAL1a‐GFP* fusion coding sequences (CDS), and inserted into the binary vector pZZ01523 to develop the *pZmNAL1a*::*ZmNAL1a*‐*GFP* construct. The CRISPR and *pZmNAL1a*::*ZmNAL1a*‐*GFP* constructs were transformed into the inbred KN5585 line via *Agrobacterium*‐mediated transformation. Gene editing alleles were screened using sgRNA target region PCR amplification and Sanger sequencing, and crossed with KN5585 inbred plants to segregate the Cas9‐free edited plants and wild‐type siblings as controls. The guide RNA sequences and PCR primers used for *ZmNAL1a*, *ZmNAL1b*, and *ZmEREBP147* genotyping are listed in Table S7.

For phenotypic evaluation, these lines were grown under a randomized block design in field locations in Wuhan (30°N, 114°E), Sanya (18°N, 109°E), and Zhangye (39°N, 100°E), with a low‐density plating of 20–25 cm between plants in the same row. The rows were planted in pairs, with approximately 50 cm between rows and 120 cm between pairs of rows. At least 15 plants were selected to score agronomic traits, including plant height, ear height and length, DTT, DTA, days‐to‐silking (DTS), total leaf and internode numbers, leaf length and leaf width, ear weight, and kernel number per row. Significant differences were examined using *P* < 0.05, one‐way ANOVA, and Tukey's HSD test.

### 4.2 Construction of the Multi‐Omics Integrative Network for NAL1‐Related Trypsin Family Genes and Flowering Time Genes

To explore the potential interaction landscape between NAL1 and flowering‐related regulators in maize, integrative network data were retrieved from the MaizeNetome database (http://minteractome.ncpgr.cn/index.php) [33]. Four NAL1 related genes (Zm00001d026585, Zm00001d001884, Zm00001d026296, and Zm00001d002323) and nine known flowering genes (ZFL1 (*Zea mays* FLORICAULA/LEAFY 1), DLF1 (Delayed flowering 1), ZMM15 (MADS‐box protein 15), ZMM4 (MADS‐box protein 4), ZAP1 (*Zea mays* apetala homolog1), ZmMADS1 (MADS‐box protein 1), ZmGI1 (GIGANTEA1), D8 (Dwarf plant 8), and D9 (Dwarf plant 9)) were used as query nodes. The minimum interaction confidence (weight) was set to > 0.3 to ensure high‐quality associations. The resulting interaction network was visualized using Cytoscape (version 3.8.2), and network clusters were manually adjusted for clarity.

### 4.3 Association Analysis of the four NAL1‐Related Trypsin Family Genes

Association analysis of the four NAL1‐related trypsin family genes was conducted in a maize‐associated panel of 540 diverse inbred lines. The SNPs in this association panel were downloaded from a previously released genotypic dataset [50]. The best linear unbiased predictions for each line across the five environments (Ya'an (30°N, 103°E), Sanya (18°N, 109°E), and Kunming (25°N, 102°E) in 2009, and Wuhan (30°N, 114°E) and Kunming (25°N, 102°E) in 2010) were calculated and used to evaluate flowering time variation. Association mapping was performed using a mixed linear model, considering population structure and relative kinship, in TASSEL v. 3.0.67 [51, 52]. Pairwise linkage disequilibrium was calculated and plotted using R software v. 3.5.1 [53]. A Bonferroni‐corrected significance threshold (*P*‐value < 0.001) was used to identify significant associations.

### 4.4 Subcellular Localization

The full‐length CDS of *ZmNAL1a* and *ZmNAL1b* were cloned and inserted into the pM999‐GFP vector upstream of GFP. Plasmids were transformed into maize leaf protoplasts extracted from 14‐day‐old etiolated seedlings using a polyethylene glycol‐mediated method [54]. Transformed protoplasts were cultured overnight in the dark at room temperature. The fluorescence was measured using a laser confocal microscope (LSM980; Zeiss).

### 4.5 Histological Observation and RNA In Situ Hybridization

For GFP localization, *proZmNAL1a::ZmNAL1a‐GFP* and *proZmNAL1a::ZmNAL1a‐GFP*;*zmnal1a‐1* complementary plants were embedded in 7% agarose (Roche, Basel, Switzerland) at 4°C for 15 min, and the shoots were transversally and longitudinally sectioned (50 µm) using a vibrating‐blade microtome (VT1200; Leica, Wetzlar, Germany) and suspended in a drop of water on a covered glass slide. The sections were observed using a confocal laser scanning microscope (LSM980; Zeiss) excited with a 488 nm argon laser, and emission images were captured at 500–560 nm. The size of the SAM area with the GFP signal was measured using ImageJ software.

For the histological analysis of SAM, shoot tips were dissected from 14, 16, 18, 20, 22, 24, 26, 28 DAP (days after planting) seedlings, fixed in ice‐cold 10% formaldehyde, 45% ethanol, and 5% acetic acid overnight, dehydrated with ethanol, and passed through 50% and 100% methyl salicylate for 1 h. The cleared shoot tips were imaged using a Nikon ECLIPSE DIC microscope (Nikon, Tokyo, Japan). SAM sizes were determined as previously described [55]. Statistical analyses were performed using a box‐and‐whisker plot in GraphPad Prism 8 software (La Jolla, CA, USA). The *P*‐values were calculated using two‐tailed paired Student's *t*‐tests. The 14 to 36 DAP seedlings were collected every two days to dissect the shoot apex for imaging SAM or IM under the stereomicroscope (Nikon, Tokyo, Japan).

For RNA in situ hybridization, shoot tips from 18 DAP seedlings from the B73 and *proZmNAL1a::ZmNAL1a‐GFP* transgenic line were used as described previously [56]. PCR amplification of the full‐length CDS and part of the 3′ untranslated regions of *ZmNAL1a*, *REL2*, and *ZmEREBP147* was used as transcription templates. Primers used for amplification are listed in Table S7. Digoxigenin‐labeled antisense probes were transcribed using an in vitro transcription kit (Roche) according to the manufacturer's instructions. The hybridized tissues were imaged using a Nikon ECLIPSE DIC microscope (Nikon).

### 4.6 Transient Expression Assays

The full‐length CDS of *ZmNAL1a* was cloned into a PFGC‐GFP vector. The constructs were transformed into *Agrobacterium* GV3101 and infiltrated into tobacco leaves for transient expression. After 72 h, the tobacco leaves were injected with 0.4 mm FM4‐64 (HY‐103466; MedChemExpress, Monmouth Junction, NJ, USA), which was used as a cell membrane marker. Fluorescence was captured at different time points using a confocal microscope (LSM980; Zeiss). Plasmodesmata were visualized by staining with aniline blue. To analyze the co‐localization of ZmNAL1a‐GFP and aniline blue signals in cell walls, the fluorescence intensity of the GFP images was adjusted so that the fluorescence in the empty regions of the cell wall was close to zero. GFP and aniline blue images were denoised using the Despeckle tool. A line was then selected for the cell wall using the segmented line tool, and the Plot Profile tool was used to quantify the fluorescence intensities of GFP and aniline blue depending on the position along the wall. The total number of peaks in the aniline blue signal was called the number of plasmodesmata, and the peaks that overlapped with the GFP peaks were determined to be plasmodesmata with ZmNAL1a accumulation [5].

### 4.7 Immunoprecipitation‐Mass Spectrometry

IP‐MS was performed to identify ZmNAL1a‐interacting proteins. Shoot apex tissues from 18‐day‐old *proZmNAL1a::ZmNAL1a‐GFP* and negative control (non‐transgenic siblings) seedlings were collected and ground in liquid nitrogen. Total proteins were extracted using a plant protein extraction kit according to the manufacturer's instructions (BB‐3124; Bestbio). The total extracted protein was precipitated using anti‐GFP magnetic agarose (D153‐10; MBL) and washed five times with phosphate‐buffered saline (PBS; 3 mm Na_2_HPO_4_, 155 mm NaCl, 1 mm KH_2_PO_4_, pH 7.4, 1 × complete protease inhibitor cocktail). The beads were added to the loading buffer, boiled for 10 min, and subjected to sodium dodecyl‐sulfate polyacrylamide gel electrophoresis (SDS‐PAGE). After in‐gel digestion, the samples were subjected to mass spectrometry (nano LC‐QEXACTIVE; Thermo Fisher Scientific, Waltham, MA, USA). Information on the proteins interacting with ZmNAL1a is shown in Table S3.

### 4.8 Y2H Assays

The CDS was removed from the self‐activated *ZmNAL1a* region, the full‐length CDS of *REL2* was cloned into the bait vector pGBKT7 (BD), and the full‐length *REL2* and *ZmEREBP147* CDS were cloned into the prey vector pGADT7 (AD) as required. Bait and prey, positive control (pGBKT7‐53 and pGADT7‐T), and negative control vectors (pGBKT7‐lam and pGADT7‐T) were transformed into the yeast strain Y2H Gold (Clontech Laboratories, Mountain View, CA, USA). The positive colonies were transferred to different growth media (SD‐Trp/‐Leu, SD‐Trp/‐Leu/‐His/‐Ade). Primers used for the Y2H assay are listed in Table S7.

### 4.9 Bimolecular Fluorescence Complementation Assays

The full‐length CDS of *ZmEREBP147*, *ZmHDT102*, and selected candidate interactors from IP‐MS were cloned into vector pXY104 for C‐terminal YFP fusion, whereas the full‐length CDS of *ZmNAL1a* and *REL2* were cloned into vector pXY106 for N‐terminal YFP fusion. The constructs were then transformed into *Agrobacteria* GV3101. Different combinations were transiently expressed in tobacco leaves. After 48 h, fluorescence was captured using a confocal microscope (LSM980; Zeiss).

### 4.10 Luciferase Complementation Imaging Assays

The full‐length *ZmHDT102* and *REL2* CDS were cloned into JW771 and JW772 [57], respectively, and transformed into *Agrobacteria* GV3101. Primers used are listed in Table S7. Cells were resuspended in infiltration buffer (10 mm MES‐K, 10 mm MgCl_2,_ and 100 µm acetosyringone, pH 5.6) when OD_600_ reached 0.8 and infiltrated into 3‐week‐old tobacco leaves. After 48–60 h, the leaves were stained with 1 mm luciferin (Promega, Madison, WI, USA), and the luciferase signal was observed using a Lumazone Pylon 2048 B imaging system.

### 4.11 Co‐Immunoprecipitation Assays

ZmNAL1a‐GFP and REL2‐HA were co‐transformed into tobacco leaves for co‐immunoprecipitation assays. At 72 h after transformation, the leaves were collected, and total proteins were extracted using a plant protein extraction kit according to the manufacturer's instructions (BB‐3124; Bestbio). Approximately 3 g of leaf tissue and 100 µL of GFP magnetic beads (D153‐10; MBL) were used for immunoprecipitation. The precipitated proteins were subjected to immunoblotting using anti‐GFP (ab290, 1:1,000; Abcam, Cambridge, UK) and anti‐HA (AE008, 1:5,000; ABclonal, Woburn, MA, USA) antibodies. Anti‐mouse IgG horseradish peroxidase (HRP)‐conjugated (AS003, 1:10 000; ABclonal) secondary antibodies were used.

### 4.12 In Vitro Pull‐Down Assays

The CDSs of *ZmNAL1a* and *REL2* were cloned into pET‐21b to generate His‐tagged fusion proteins (ZmNAL1a‐His and REL2‐His), while *REL2*, *ZmEREBP147*, and *ZmHDT102* CDSs were cloned into pGEX‐4T‐1 to produce GST‐tagged fusions (REL2‐GST, ZmEREBP147‐GST, and ZmHDT102‐GST). For each pull‐down assay, 1 µg of one of the following recombinant protein pairs (REL2‐GST + ZmNAL1a‐His, ZmEREBP147‐GST + REL2‐His, or ZmHDT102‐GST + ZmNAL1a‐His) was incubated in 50 µL of GST beads (C650031; Sangon Biotech, Shanghai, China) in a GST‐binding buffer (20 mm HEPES, 40 mm KCl, 1 mm EDTA; pH 7.5). The mixture was washed three times with GST wash buffer (50 mm Tris‐HCl, 150 mm NaCl, 0.1% Triton X‐100; pH 8.0) for 10 min. The cleared proteins were eluted by boiling in 1× SDS loading buffer, separated by SDS‐PAGE, and detected using anti‐GST (AE001, 1:5,000; ABclonal) and anti‐His antibodies (AE003, 1:5,000; ABclonal).

### 4.13 Cell‐Free Protein Degradation Assays

A cell‐free protein degradation assay was performed as previously described [58]. Total protein was extracted from WT and *zmnal1a* seedlings using a degradation buffer (25 mm Tris‐HCl, pH 7.5, 10 mm NaCl, 10 mm MgCl_2_, 10 mm ATP). After two 10‐min centrifugations at 16 000 × *g* and 4 °C, the supernatant was collected, and the protein concentration was determined by absorbance at 280 nm using a NanoDrop 2000 Spectrophotometer (Thermo Fisher Scientific). Each cell‐free degradation assay was performed in 250 µL of degradation buffer, including 500 µg of total proteins and 100 ng of purified REL2‐GST or ZmHDT102‐GST. Total protein extracts from WT plants were used for the protease inhibitor treatments using serine protease inhibitor (5 mm PMSF). The mixture was incubated at 37 °C. An equal volume of the protein mixture was collected at 0, 15, 30, and 60 min and subjected to western blotting using an anti‐GST antibody (AE001, 1:5,000; ABclonal). Actin was used as the loading control. Mouse anti‐mouse IgG (AS003, 1:10 000; ABclonal) was used as the secondary antibody.

### 4.14 In Vivo Protein Degradation Assays

A total of 30 µg of REL2‐FLAG, ZmHDT102‐FLAG, or GFP plasmids were transfected into WT and *zmnal1a‐1* protoplasts. After 16 h incubation, the protoplasts were treated with 200 µm protein synthesis inhibitor cycloheximide (01810; Sigma–Aldrich, St. Louis, MO, USA) and mixed gently. Equivalent amounts of protoplasts were collected at 0, 1, 3, and 5 h. Total protein was extracted with an extraction buffer (50 mm Tris‐HCl, pH 7.5, 150 mm NaCl, 10% glycerol, 0.1% IGEPAL CA‐630, 1× complete protease inhibitor cocktail) and subjected to western blotting with anti‐Flag (AE005, 1:5,000; ABclonal) and anti‐GFP (ab290, 1:1,000; Abcam) antibodies. GFP was used as a negative control, and anti‐mouse IgG was used as the secondary antibody (AS003, 1:10 000; ABclonal).

### 4.15 In Vitro Protein Degradation Assays

The proteolytic activity of ZmNAL1a to REL2 and ZmHDT102 was assayed in reaction buffer (25 mm Tris‐HCl, pH 8.0, 10 mm CaCl_2_, 2 mm ATP) containing 0.2 mg of purified ZmNAL1a‐GST or GST proteins and 0.2 mg of purified REL2‐GST or ZmHDT102‐GST protein in a total volume of 200 µL. The mixture was incubated at 37 °C. An equal volume of the protein mixture was collected at 0, 30, 60, and 90 min and subjected to western blotting with anti‐GST antibodies. GST (AE001, 1:5,000; ABclonal) was used as a negative control, and anti‐mouse IgG (AS003, 1:10 000; ABclonal) was used as a secondary antibody.

### 4.16 RNA‐Seq Analysis

The SAM tissues were collected from 18 DAP seedlings in WT and *zmnal1a‐1* mutants. Total RNA was extracted from these SAM tissues using TRIzol Reagent (Life Technologies, Invitrogen, Carlsbad, CA, USA) according to the manufacturer's instructions, followed by cleanup and DNase I treatment using an RNeasy Mini Kit (Qiagen, Hilden, Germany). Three independent biological replicates were used for each plant. mRNA sequencing libraries were prepared according to the manufacturer's instructions (TruSeq Standard RNA LT Guide) and sequenced on the Illumina Nova platform (Illumina, San Diego, CA, USA) at Beijing Novogene. Effective reads were aligned to the maize reference genome (AGPv4) using HISAT2 v. 2.1.0 [59]. Differential gene expression was analyzed using Cufflinks v2.2.1 [60]. DEGs were defined by false discovery rate (FDR) < 0.05 and fold change > 1.5. Gene Ontology analysis was performed using Agrigo software v. 2.0 [61].

### 4.17 Chromatin IP Assays and Data Analysis

The ChIP experiments were performed as previously described [62, 63]. Briefly, approximately 2 g of SAM‐enriched tissue from 18 DAP seedlings was dissected and cross‐linked in 30 mL of 1× PBS (137 mm NaCl, 2.7 mm KCl, 10 mm Na_2_HPO_4_, 2 mm KH_2_PO_4_) containing 1% formaldehyde for 15 min under vacuum. Cross‐linking was terminated by adding 0.15 m glycine, followed by a 5 min incubation under vacuum. The samples were washed three times with distilled water, dried with paper towels, and immediately frozen in liquid nitrogen. Chromatin was extracted with extraction buffer 1 (EB1, 0.4 m sucrose, 10 mm Tris‐HCl, pH 8.0, 10 mm MgCl_2_, 5 mm mercaptoethanol, plant protease inhibitor cocktail). The sample was centrifuged at 1,000 × *g* for 20 min at 4 °C. The pellet was washed five times with 5 mL of EB2 (0.25 m sucrose, 10 mm Tris‐HCl, pH 8.0, 10 mm MgCl_2_, 1% Triton X‐100, 5 mm mercaptoethanol, plant protease inhibitor cocktail) and once with 5 mL of EB3 (1.7 m sucrose, 10 mm Tris‐HCl, pH 8.0, 2 mm MgCl_2_, 0.15% Triton X‐100, 5 mm mercaptoethanol, plant protease inhibitor cocktail), followed by sonication in 300 µL of sonication buffer (50 mm Tris‐HCl, pH 8.0, 10 mm EDTA, 1% SDS, plant protease inhibitor cocktail) using a Diagenode Bioruptor sonicator (Diagenode SA, Seraing, Belgium) for 15 cycles (30 s ON/30 s OFF). The sonicated sample was centrifuged for 10 min at 12 000 × *g* and 4 °C, and the supernatant was collected and used for chromatin isolation. ChIP was performed using a specific antibody against the acetylated histone H3K9Ac (ab10812, 1:5000; Abcam). Libraries were constructed using an Ovation Low Input DR kit (NuGEN Technologies, San Carlos, CA, USA) after de‐crosslinking, isolation, and purification of the immunoprecipitated DNA. Two input and two IP libraries were sequenced on a HiSeq 2000 platform (Illumina).

ChIP‐seq reads were aligned to the maize reference genome (AGPv4) using HISAT2 v.2.1.0 [59]. Only the uniquely mapped reads were used for further processing. After the removal of PCR duplicates and low‐quality reads (mapping quality score < 10) using samtools v. 1.9.0 [63], MACS2 v. 2.1.1 was used to call peaks with default parameters [64]. ChIP tracks showing fusion protein‐binding sites were visualized using IGV [65].

### 4.18 Quantitative Real‐Time PCR Analysis

Total RNA was extracted from 18 DAP SAM tissues of WT and *CR‐erebp147* plants using TRIzol Reagent (Life Technologies) according to the manufacturer's instructions. DNase I (TaKaRa Biotech, Dalian, China) was used to remove genomic DNA contamination. Oligo (dT) primers and M‐MLV reverse transcriptase (Invitrogen) were used to synthesize first‐strand cDNA. Quantitative real‐time PCR was performed using Universal SybrGreen Master Mix (Bio‐Rad, Hercules, CA, USA) on a CFX96 Real‐Time System (Bio‐Rad). The maize Actin gene (*Zm00001d010159*) was used as the internal control. The relative expression of the gene was calculated using the 2^−ΔCt^ method. The primers used for quantitative real‐time PCR are listed in Table S7.

### 4.19 Dual Luciferase Assays

The coding sequences of *ZmEREBP147* were cloned into the pGreenII 62‐SK vector and placed under the control of the 35S CaMV promoter as an effector construct. Cloned promoter fragments upstream from the start codon of *DLF1* (4,111 bp), *ZAG6* (1,105 bp), *ZmSPL13* (2,128 bp), and *ZFL1* (1,525 bp) were cloned into the pGreenII‐0800‐LUC vector to generate another set of reporters. Appropriate combinations of reporter and effector constructs were co‐transformed into maize leaf protoplasts using the corresponding reporter with the empty effector pGreenII 62‐SK as a control. Luciferase activity was measured using the Dual‐Luciferase Reporter Gene Assay Kit (Promega) according to the manufacturer's instructions.

### 4.20 Quantification and Statistical Analyses

Quantification of fluorescent signals and immunoblotting signals was conducted using ImageJ software (https://imagej.nih.gov/ij/). All data plotting and statistical analyses were performed with GraphPad Prism 8.0 software (https://www.graphpad.com/). Details about the statistical parameters, such as the means ± SDs (standard deviations) and the number of samples (n), are shown in the figure legends. A two‐tailed Student's *t*‐test was used for significant difference analysis between two samples. One‐way ANOVA analyses followed with Tukey's honestly significant difference (HSD) test were used for pairwise multiple comparisons. Asterisks indicate statistical significance: *P < 0.05; **P < 0.01; ***P < 0.001; NS, not significant.

### 4.21 Gene Accession Number Information

The genes studied in this work are under the following accession numbers: ZmNAL1a (Zm00001d002323), ZmNAL1b (Zm00001d026296), REL2 (Zm00001d024523), ZmHDT102 (Zm00001d011139), ZmEREBP147 (Zm00001d043205), DLF1 (Zm00001d022613), ZAG6 (Zm00001d027425), ZmSPL13 (Zm00001d006451), ZFL1 (Zm00001d026231), ZmSPL29 (Zm00001d021573), ZmMADS69 (Zm00001d042315), ZmPHYB1 (Zm00001d028905), ZmSPL6 (Zm00001d042319) and Gl15 (Zm00001d046621).

## Author Contributions

N.L., Q.N., Z.Z., and L.L conceived and designed the experiment. N.L., Z.L., Q.Z., P.Z., Y.L., Q.X., and J.L. performed all experiments. Q.N. and L.D. analysed the data. J.Z. and Z.L. planted materials. N.L., Q.N., J.L., D.J., M.K., Z.Z., and L.L. wrote and revised the article.

## Conflicts of Interest

The authors declare no conflicts of interest.

## Supporting information




**Supporting File 1**: advs73613‐sup‐0001‐SuppMat.docx.

**Supporting File 2**: advs73613‐sup‐0002‐Supplementary tables‐R3.xlsx.

**Supporting File 3**: advs73613‐sup‐0003‐Supporting information‐Western Blot data.pdf.

**Supporting File 4**: advs73613‐sup‐0004‐Data.zip.

## Data Availability

RNA‐seq and ChIP‐seq data generated in this study have been deposited in the National Genomics Data Center, China National Center for Bioinformation, under accession no. CRA019710 (https://ngdc.cncb.ac.cn/gsa). Source data are provided with this paper.
