# Supplementary material for: A Trypsin‐Like Serine Protease ZmNAL1a Fine‐Tunes Maize Floral Transition and Flowering Time
Source: Adv Sci (Weinh). 2026 Jan 4;13(10):e14635. doi: 10.1002/advs.202514635 (PMC12915138; doi:10.1002/advs.202514635)

Figure. 4b

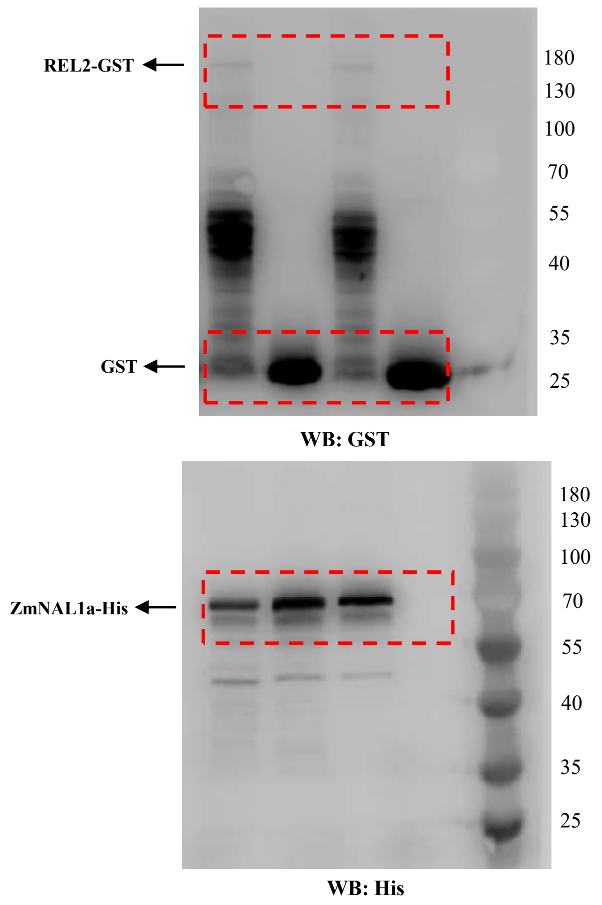

Figure. 4c

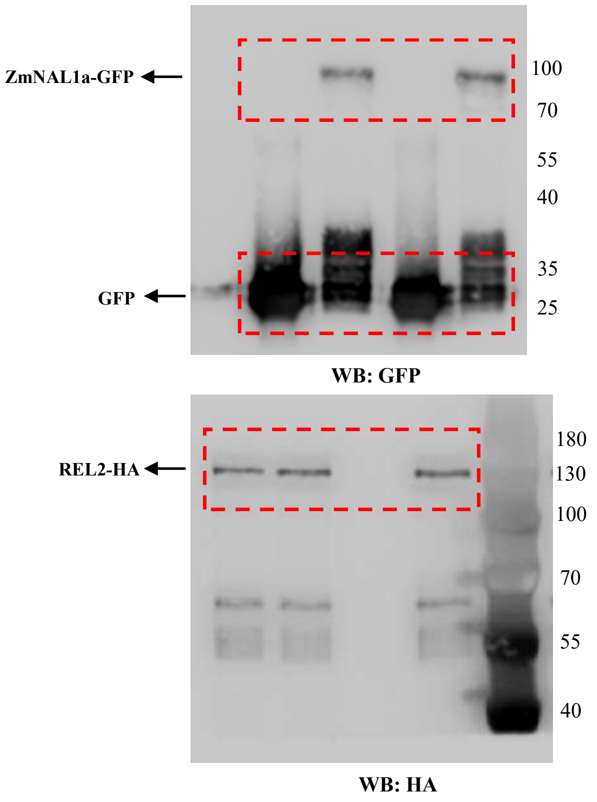

Figure. 4d

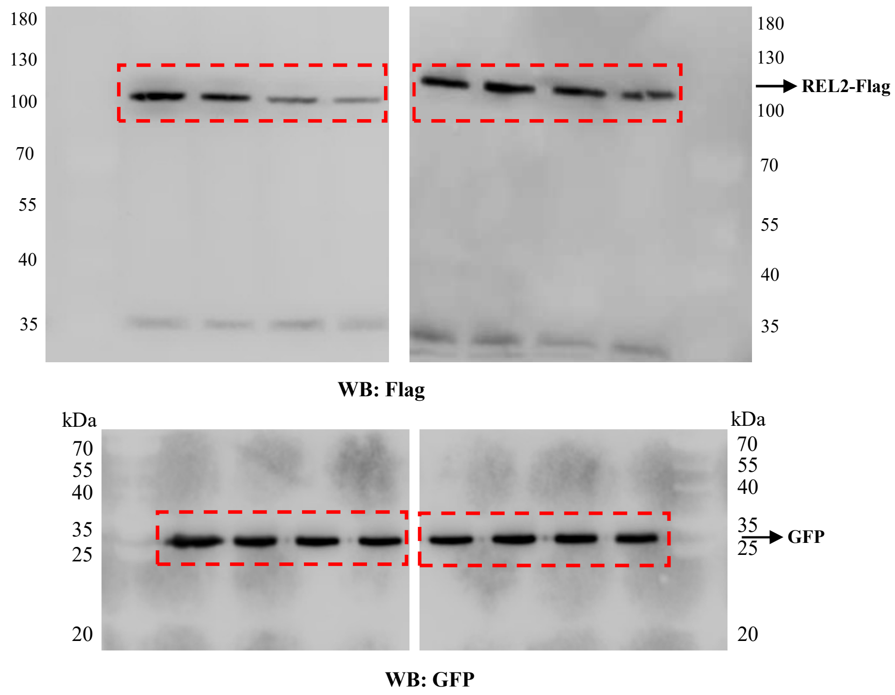

Figure. 4e

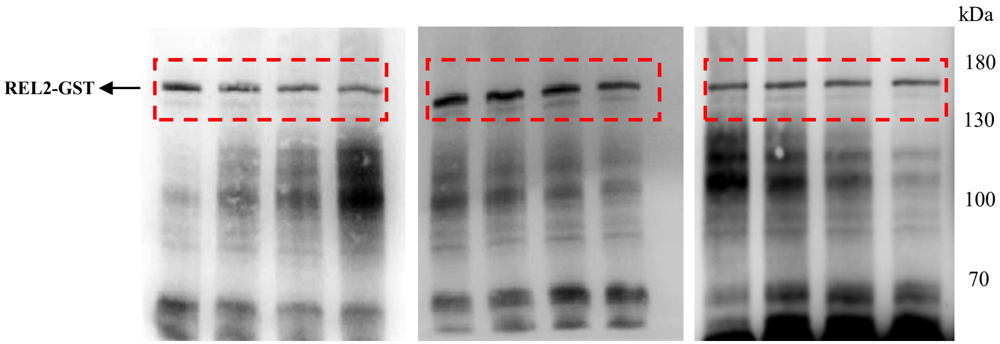

WB: GST

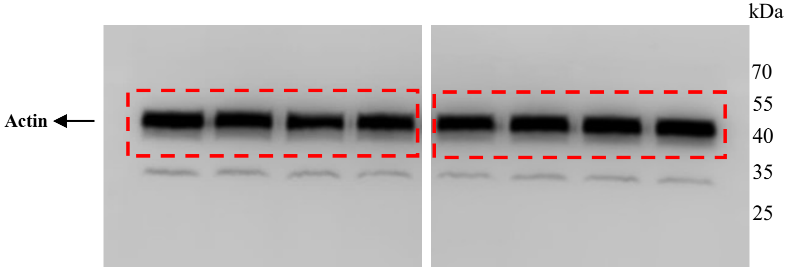

WB: Actin

Figure. 4f

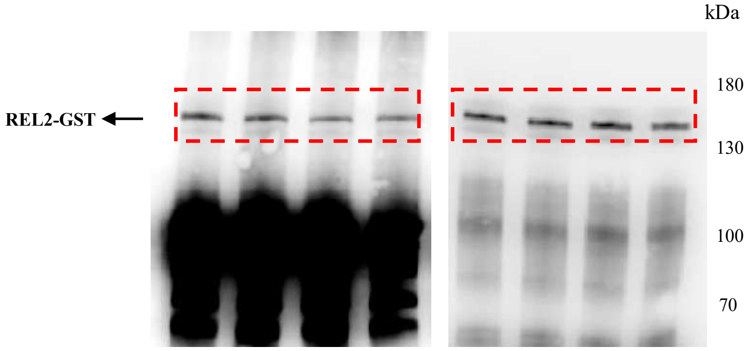

WB: GST

Figure. 6b

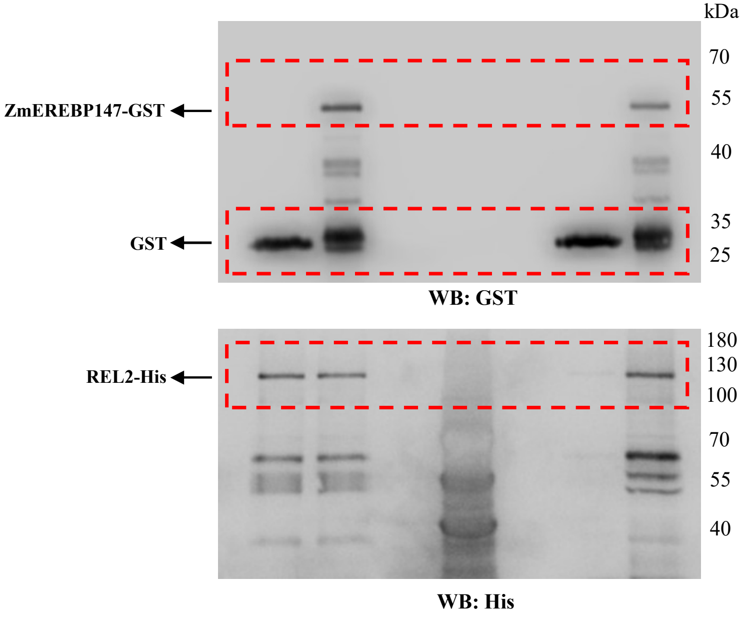
kDa
70
55
40
35
25
ZmEREBP147-GST
GST
WB: GST
180
130
100
70
55
40
REL2-His
WB: His

Figure. S18a

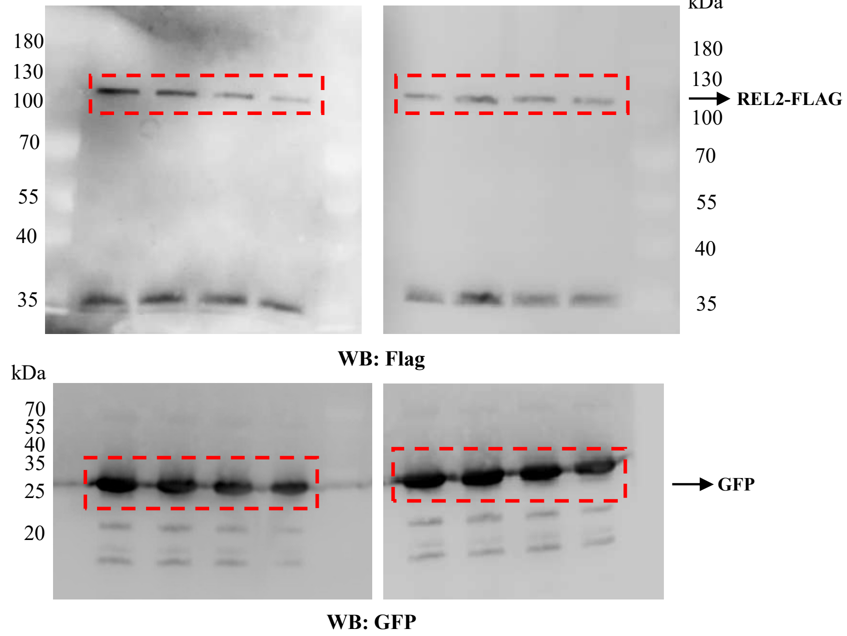

Figure. S18b

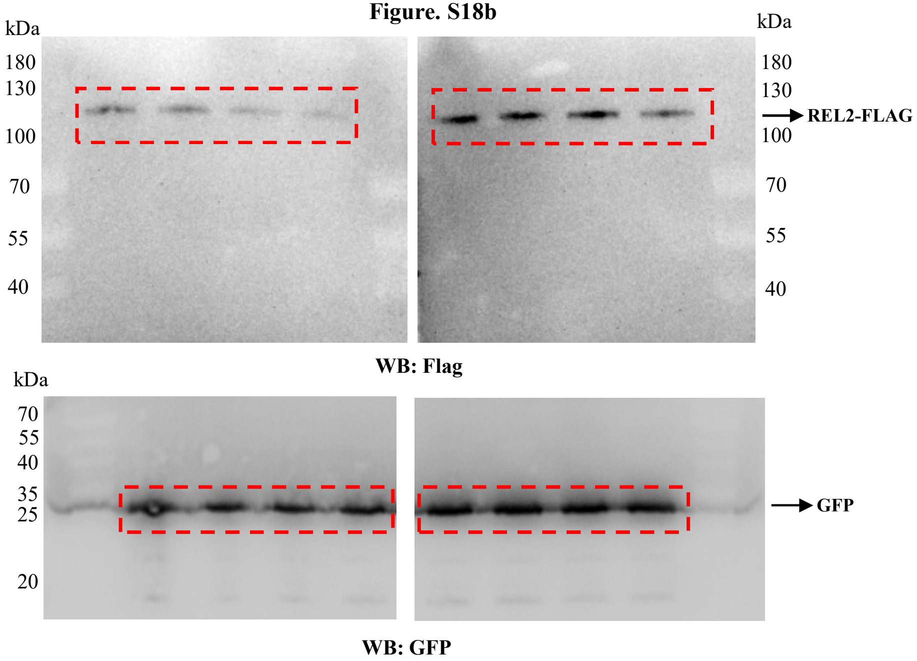

Figure. S18c

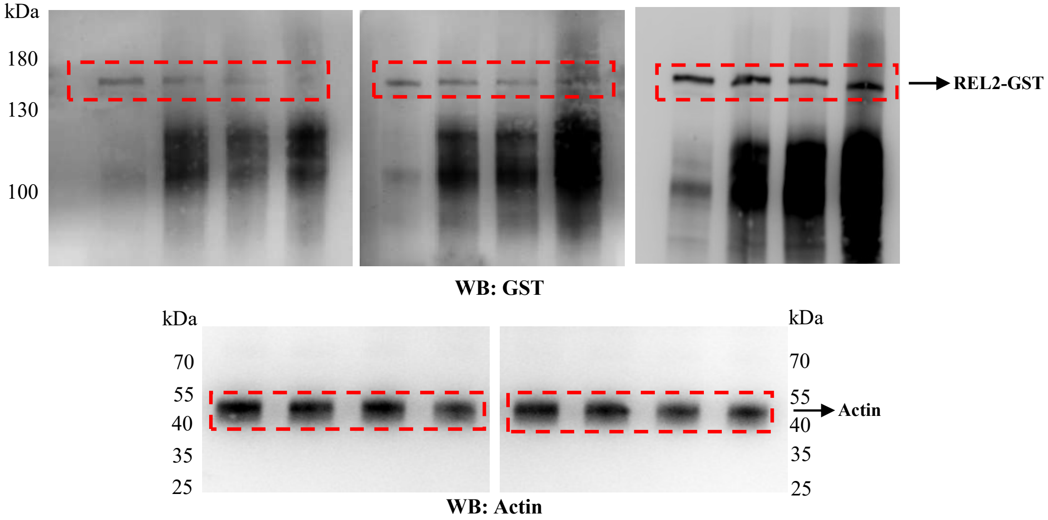

Figure. S18d

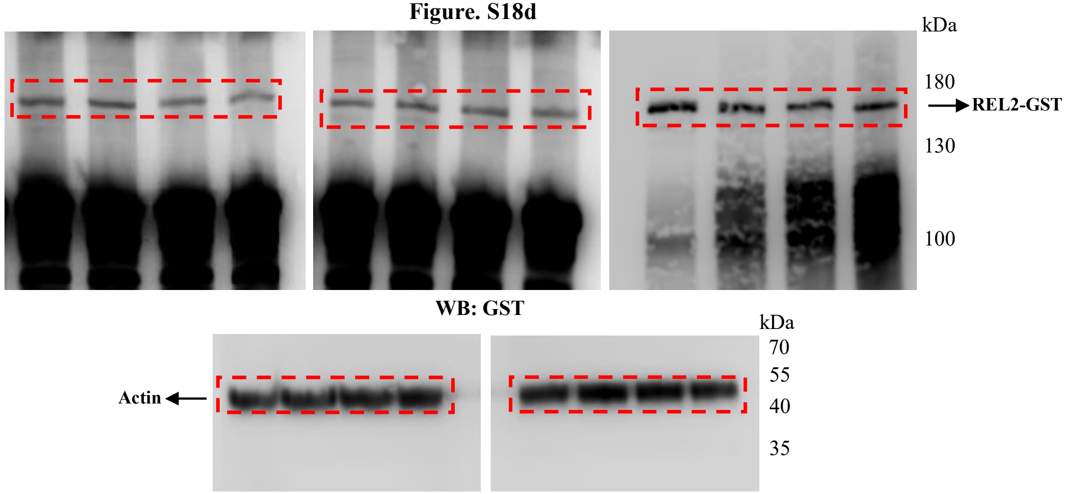

Figure. S18e

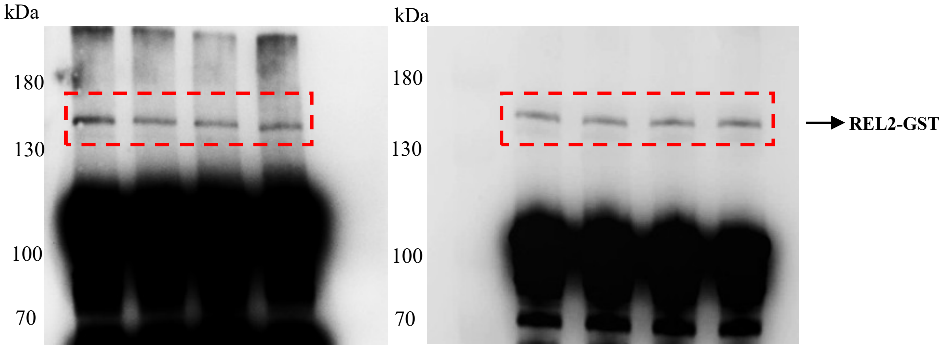

Figure. S18f

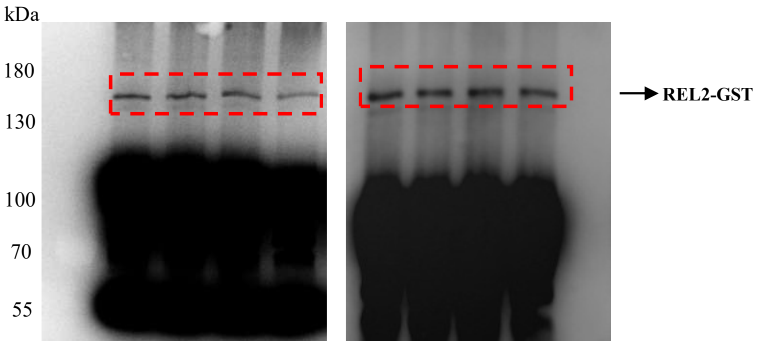

Figure. S19a

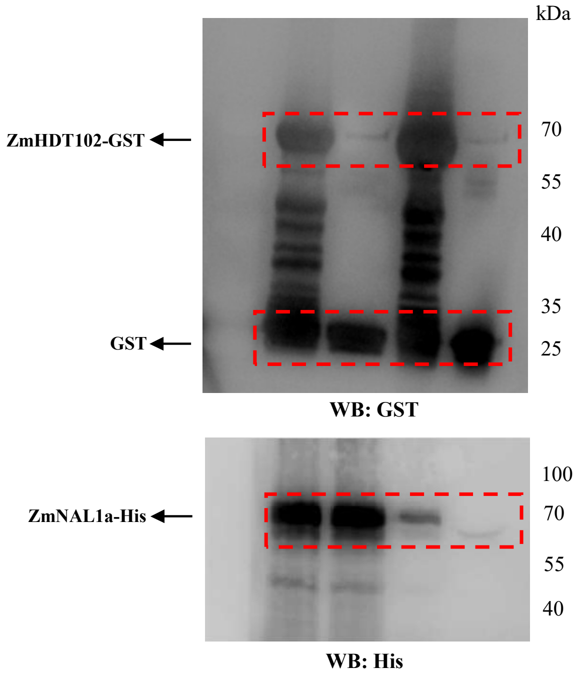

Figure. S19c

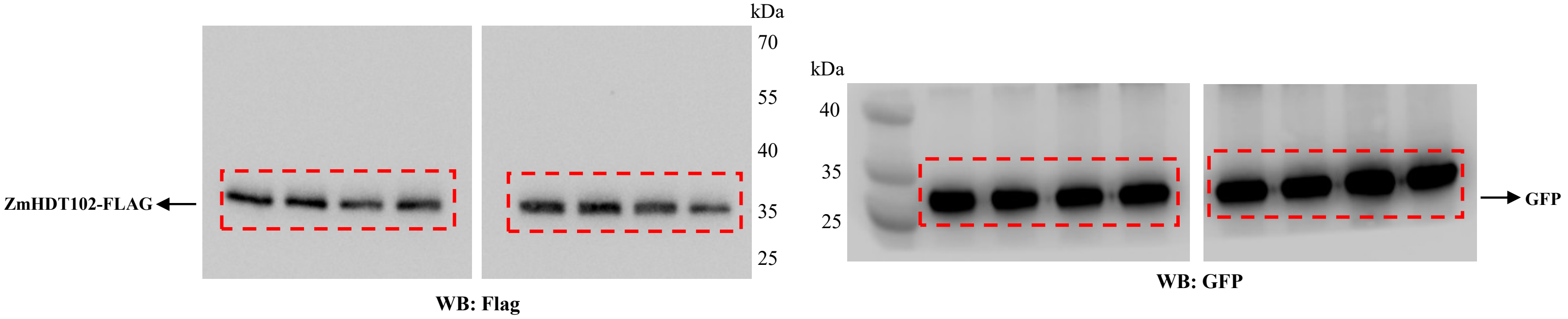

Figure. S19d

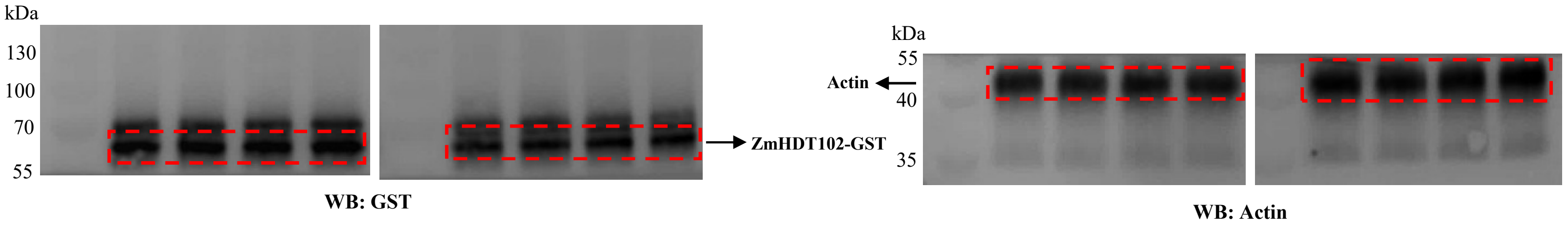

Figure. S19e

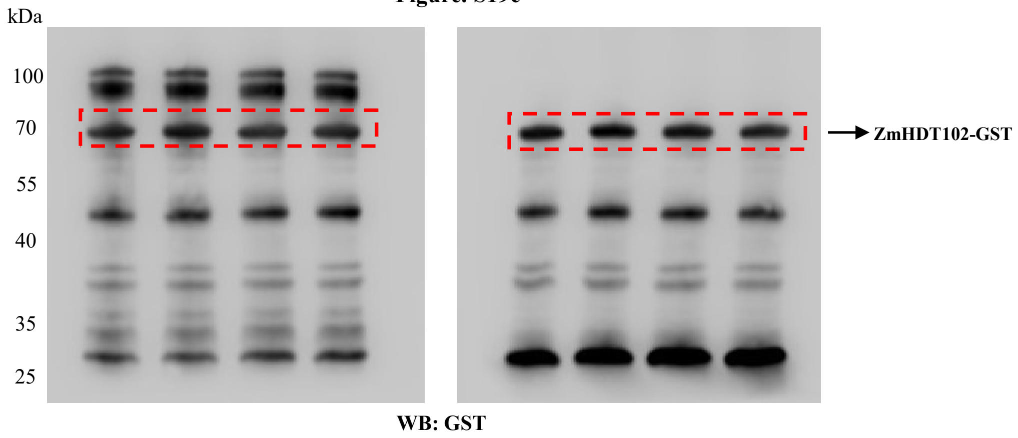

Supplement: Supplementary file 3 — Supporting File 3: advs73613‐sup‐0003‐Supporting information‐Western Blot data.pdf. [file ADVS-13-e14635-s004.pdf]
