# Supplementary material for: A Trypsin‐Like Serine Protease ZmNAL1a Fine‐Tunes Maize Floral Transition and Flowering Time
Source: Adv Sci (Weinh). 2026 Jan 4;13(10):e14635. doi: 10.1002/advs.202514635 (PMC12915138; doi:10.1002/advs.202514635)
Supplement: Supplementary file 1 — Supporting File 1: advs73613‐sup‐0001‐SuppMat.docx. [file ADVS-13-e14635-s002.docx]

Supporting Information

**A Trypsin-like Serine Protease ZmNAL1a Fine-tunes Maize Floral Transition and Flowering Time**

*Nan Li, Qiang Ning, Zhen Li, Qian Zhang, Peilu Zhu, Jimin Zhan, Yunfu Li, Zhen Li, Liang Dong, Qing Xiong, Jiahao Liao, Jie Liu, David Jackson, Munenori Kitagawa, Zuxin Zhang*, Lei Liu**

* E-mail: [leil@mail.hzau.edu.cn](mailto:zuxinzhang@mail.hzau.edu.n), [zuxinzhang@mail.hzau.edu.cn](mailto:zuxinzhang@mail.hzau.edu.n)

**This WORD file includes:**

Supplementary Figure 1 to 24

**Other Supplementary Materials for this manuscript include th following:**

Supplementary Data 1 to 7 (Excel format)

**Figure S1.** Integrated multi-omics network of four NAL1-related genes and known flowering-related genes in maize, and association analysis of the four genes with days-to-anthesis (DTA).

a) Integrative network showing associations among four NAL1 genes and nine known flowering-related genes in maize. The interaction data were retrieved from the maize interactome database (<http://minteractome.ncpgr.cn/index.php>)^[33]^ using a weight threshold of > 0.3. Nodes represent genes, and edges denote predicted or experimentally supported interactions. NAL1 proteins and nine known flowering regulators (ZFL1, DLF1, ZMM15, ZMM4, ZAP1, ZmMADS1, ZmGI1, D8, and D9) are highlighted at the center of subnetworks. b) Association between genetic variation within four NAL1 genes (*Zm00001d001884*, *Zm00001d002323*, *Zm00001d026296*, and *Zm00001d026585*) and DTA was analyzed in a panel of 540 diverse maize inbred lines. A SNP (indicated by the red arrow) in the promoter region of *Zm00001d002323* showed a significant association with DTA. The structures of the four NAL1 genes are shown below the Manhattan plots.

**Figure S2.** Phylogenetic tree of Zm00001d002323 and its homologs.

The amino acid sequences of ZmNAL1 proteins are downloaded from the public database Ensembl Plants (<http://plants.ensembl.org/index.html>). The phylogenetic tree is constructed using the maximum likelihood method with 1,000 bootstraps using MEGA7. The scale bar indicates the average number of amino acid substitutions per site. OsNAL1, ZmNAL1a, and ZmNAL1b are highlighted in red script.

**Figure S3.** Alignment of amino acid sequences of ZmNAL1a and its homologs from diverse species.

Background colors indicate the similarity level of amino acid sequences (dark blue, = 100%; pink, < 100% and ≥ 75%; baby blue, < 75% and ≥ 50%). The green line represents the trypsin-like serine proteases domain.

**Figure S4.** Candidate gene association analysis of *ZmNAL1a* indicates a variant in its promoter showing association with flowering time.

a) Association between genetic variation from *ZmNAL1a* gene body and its 20 kb flanking regions and days-to-anthesis (DTA) was studied in a panel of 540 diverse maize inbred lines. The SNP-1468 (red dot) in *ZmNAL1a* promoter was found to have a significant association with DTA. The gene structure of *ZmNAL1a* is shown below the Manhattan plot, and the position of the SNP-1468 relative to the transcription start site (TSS) is indicated. The white to black heatmap shows the pairwise linkage disequilibrium pattern by R^2^. b) At the association site SNP-1468, two haplotypes (AA and GG) were identified in the diverse panel. c-e) Box-and-whisker plots of days-to-tasseling (c), days-to-anthesis (d) and days-to-silking (e). In the diverse panel, 111 inbred lines carry haplotype AA, while 301 inbred lines are haplotype GG at the SNP-1468. Each box represents the median and interquartile range. Whiskers extend to maximum and minimum values. Two-tailed Student’s *t*-test estimates the significance of the difference.

**Figure S5.** Genotypes of *ZmNAL1a* and *ZmNAL1b* mutants.

a-b) Genotypes of *zmnal1a* (a) and *zmnal1b* (b) mutants generated by CRISPR/Cas9 technology. Black shading, exon; gray shading, untranslated region. The red line indicates the guide RNA sites.

**Figure S6.** Phenotypes of *zmnal1a* and *zmnal1b* mutants.

a) Phenotypic comparison of leaf length in wild-type (WT) (n = 24), *zmnal1b-1* (n = 21), *zmnal1a-1* (n = 34), and *zmnal1a-1*;*zmnal1b-1* (n = 25). b-d) Phenotypic comparison of internode number (b), plant height (c) and ear height (d) in WT (n = 23), *zmnal1b-1* (n = 23), *zmnal1a-1* (n = 34), and *zmnal1a-1*;*zmnal1b-1* (n = 28). e-f) Phenotypic comparison of ear length (e) and kernel number per row (f) in WT (n = 13), *zmnal1b-1* (n = 15), *zmnal1a-1* (n = 20), and *zmnal1a-1*;*zmnal1b-1* (n = 21). Data are presented as mean values ± s.d., and different lowercase letters above at the top of each bar indicate significant groupings (*P* < 0.05, one-way ANOVA, Tukey’s HSD test, Table S2, Supporting Information). Source data are provided as a Source Data file.

**Figure S7.** Knockout mutants of *ZmNAL1a* show delayed flowering.

a) Plant morphologies of wild-type (WT), *zmnal1a-1* and *zmnal1a-2* knockout mutants. Arrowheads indicate the ears. Scale bars, 20 cm. b-d) Phenotypic comparison of days-to-tasseling (b), days-to-anthesis (c) and days-to-silking (d) between WT (n = 92), *zmnal1a-1* (n = 59) and *zmnal1a-2* (n = 50) knockout mutants. Data are presented as mean values ± s.d., and different lowercase letters above at the top of each bar indicate significant groupings (*P* < 0.05, one-way ANOVA, Tukey’s HSD test, Table S2, Supporting Information). Source data are provided as a Source Data file.

**Figure S8.** Knockout mutants of *ZmNAL1b* show no significant difference in flowering time compared to the wild type.

a) Plants of wild-type (WT) and *zmnal1b* knockout lines. Arrowheads indicate the ears. Scale bars, 20 cm. b-d) Phenotypic comparison of days-to-tasseling (b), days-to-anthesis (c) and days-to-silking (d) between WT (n = 44), *zmnal1b-1* (n = 51) and *zmnal1b-2* (n = 44) knockout lines. Data are presented as mean values ± s.d., and different lowercase letters above at the top of each bar indicate significant groupings (*P* < 0.05, one-way ANOVA, Tukey’s HSD test, Table S2, Supporting Information). Source data are provided as a Source Data file.

**Figure S9.** *zmnal1a-1* shows the reduced size of shoot apical meristem (SAM) compared to wild type siblings.

Diameter of SAM was quantified. Vegetative growth stages (V stages) were defined according to the full extension of the leaf collar of the uppermost leaf. Sample size n ≥ 8. Data are means ± s.d. Asterisks indicate significance of difference, and ns indicates no significant difference between WT and the *zmnal1a*-*1* mutant by two-tailed Student’s *t* test. **, *P* < 0.01; ***, *P* < 0.001. Exact *P* values were listed in Table S2, Supporting Information. Source data are provided as a Source Data file.

**Figure S10.** Dynamic observation of the transition from vegetative to reproductive development in SAM transition.

Representative images of SAM from WT and *zmnal1-1* mutant plants at 14 to 36 days after planting. Vegetative growth stages (V stages) were defined according to the full extension of the leaf collar of the uppermost leaf. Scale bars, 200 μm.

**Figure S11.** Expression pattern and subcellular localization of ZmNAL1a.

a) Expression patterns of *ZmNAL1a* in distinct tissues. Expression data were retrieved from the qTeller database (https://qteller.maizegdb.org). Vegetative meristem (16-19 days) is a mixture of SAM and leaf primordia. FPKM, Fragments Per Kilobase of exon model per Million mapped fragments. b) ZmNAL1a proteins were localized in the nucleus and cytoplasm of maize leaf protoplasts. Scale bars, 20 μm. c-d) Distribution of ZmNAL1a-GFP fusion proteins expressed in leaves of *proZmNAL1a::ZmNAL1a-GFP* plants. The red arrowheads indicate ZmNAL1a-GFP droplets. Scale bars, 20 μm.

**Figure S12.** mRNA in situ hybridization pattern of *ZmNAL1a* in SAM.

a) Wild-type SAM hybridized with a *ZmNAL1a* sense probe (negative control). b) *ProZmNAL1a*::*ZmNAL1a*-*GFP* SAM hybridized with a GFP sense probe (negative control). c-d) Wild-type SAM hybridized with *KN1* (c) and *DRL2* (d) antisense probes as positive controls. e-h) RNA in situ hybridization of *ZmNAL1a-GFP* in the SAM of *proZmNAL1a::ZmNAL1a-GFP* plants at different vegetative growth stages using a GFP antisense probe. Vegetative growth stages (V stages) were defined according to the full extension of the leaf collar of the uppermost leaf. Scale bars, 100 μm.

**Figure S13.** The genetic complement assay revealed that the *proZmNAL1a::ZmNAL1a-GFP* transgenic could largely recover the delayed flowering phenotype of *zmnal1a-1*.

a) Morphologies of wild-type (WT), *proZmNAL1a*::*ZmNAL1a-GFP*;*zmnal1a-1* complementary line and *zmnal1a-1* mutant. Scale bars, 10 cm. b-d) Phenotypic analysis of days-to-tasseling (b), days-to-anthesis (c) and days-to-silking (d) in the WT (n = 26, 29, 28), *proZmNAL1a*::*ZmNAL1a-GFP*;*zmnal1a-1* complementary line (n = 41, 37, 37) and *zmnala-1* mutants (n = 48, 48, 42). Data are presented as the mean values ± s.d., and different lowercase letters indicate significant groupings (*P <* 0.05, one-way ANOVA, Tukey’s HSD test, Table S2, Supporting Information). Source data are provided as a Source Data file.

**Figure S14.** ZmNAL1a protein forms punctate structures in the cytoplasm and undergoes transmembrane movement.

a, f, k, p and u are representative confocal images showing the subcellular localization of ZmNAL1a in the different tobacco leaf epidermal cells. b-e, g-j, l-o, q-t and v-y are time-lapse images of the boxed region enlarged image of (a), (f), (k), (p) and (u), respectively. Green, GFP; Red, FM4-64. White triangles indicate the mobile puncta of the ZmNAL1a-GFP protein. White arrows indicate the movement of the puncta. Scale bars, 10 μm.

**Figure S15.** Localization of GFP alone and FM4-64 in tobacco leaf cells.

GFP alone was injected into tobacco leaf epidermal cells, and no punctate structures were found in the cytoplasm. Scale bars, 50 μm.

**Figure S16.** BiFC validation of ZmNAL1a interactions with a subset of proteins identified by IP-MS. Scale bars, 50 μm.

**Figure S17.** ZmNAL1a physically interacts with REL2.

Schematic representation of the domain constructs transformed into yeast (left panel) and yeast-two hybrid assay (right panel). The ZmNAL1a-REL2 interaction is dependent on the C-terminal EAR1 motif.

**Figure S18.** Repeat of the validation of ZmNAL1a-mediated REL2 degradation.

a-b) In vivo degradation assays of ZmNAL1a-mediated REL2 degradation performed in protoplasts. c-d) Cell-free protein degradation assays using total plant extracts. e-f) In vitro protein degradation assays with purified proteins. Consistent results were obtained from independently repeated experiments.

**Figure S19.** ZmNAL1a interacts with ZmHDT102 but does not degrade it.

a-b) The physical interaction between ZmNAL1a and ZmHDT102 was verified by pull-down assays (a) and BiFC assays (b). Scale bars, 20 μm. c-e) In vivo protein degradation (c), cell-free protein degradation (d) and in vitro protein degradation (e) assays demonstrate that ZmNAL1a does not degrade ZmHDT102.

**Figure S20.** REL2 physically interacts with ZmHDT102.

a-b) Physical interaction between REL2 and ZmHDT102 was verified by BiFC assays (a) and LCI assays (b) in tobacco leaf. Scale bars, 20 μm.

**Figure S21.** Gene ontology (GO) enrichment analysis for the differentially expressed genes.

a) Gene ontology (GO) enrichment analysis of upregulated genes in shoot apex meristems of the *zmnal1a* compared to wild type. b) GO enrichment of downregulated genes in shoot apex meristems of the *zmnal1a* compared to wild type. The color of the points corresponds to the value of -log_10_(*P*-value). The size of each point corresponds to the gene number of each GO term.

**Figure S22.** mRNA in situ hybridization using antisense *ZmEREBP147* (a) and *REL2* (c) probes in the SAM, with sense probes (b, d) as controls. Scale bars, 50 μm.

**Figure S23.** Genotype of *CR-zmerebp147* mutant alleles.

Knockout mutants of *ZmEREBP147* were generated by CRISPR/Cas9 genome editing. Gene diagram is shown. Black shading, exon. The red line at the exon indicates the target site of guide RNA.

**Figure S24.** *CR-zmerebp147* could induce an earlier floral transition compared to wild type siblings.

a) Images show cleared SAMs in the WT and *CR-zmerebp147-2* mutants at 14, 16, 18, 20, 22, 24, 26, and 28 days after planting, and solid lines indicate the meristem height and diameter. Scale bars, 100 μm. b-c) SAM height (b) and diameter (c) of WT and *CR-zmerebp147-2* mutants (n ≥ 6). Vegetative growth stages (V stages) were defined according to the full extension of the leaf collar of the uppermost leaf. Data are means ± s.d. Asterisks indicate significance of difference, and ns indicates no significant difference between WT and the *CR-zmerebp147-2* mutant by two-tailed Student’s *t* test. **, *P* < 0.01; ***, *P* < 0.001. Exact *P* values were listed in Table S2, Supporting Information. Source data are provided as a Source Data file.
